# An oncogenic KRAS transcription program activates the RHOGEF *ARHGEF2* to mediate transformed phenotypes in pancreatic cancer

**DOI:** 10.18632/oncotarget.13152

**Published:** 2016-11-07

**Authors:** Oliver A. Kent, María-José Sandí, Helen E. Burston, Kevin R. Brown, Robert Rottapel

**Affiliations:** ^1^ Princess Margaret Cancer Centre, University Health Network, Toronto Medical Discovery Tower, University of Toronto, Toronto, Canada; ^2^ Donnelly Centre and Banting and Best Department of Medical Research, University of Toronto, Toronto, Canada; ^3^ Department of Medicine, St. Michael's Hospital, Toronto, Canada; ^4^ Department of Medical Biophysics, St. Michael's Hospital, Toronto, Canada; ^5^ Department of Immunology, St. Michael's Hospital, Toronto, Canada; ^6^ Division of Rheumatology, St. Michael's Hospital, Toronto, Canada

**Keywords:** KRAS, ARHGEF2, GEFH1, transcription, pancreatic

## Abstract

Activating mutations of KRAS are nearly ubiquitous in pancreatic adenocarcinomas occurring in greater than 90% of cases. Cellular transformation by oncogenic RAS requires the RHO guanine exchange factor *ARHGEF2* (also known as GEF-H1) for tumor growth and survival. Here, we find oncogenic KRAS activates *ARHGEF2* through a minimal RAS responsive promoter. We have determined the endogenous *ARHGEF2* promoter is positively regulated by the transcription factors ELK1, ETS1, SP1 and SP3 and negatively regulated by the RAS responsive element binding protein (RREB1). We find that the panel of *ARHGEF2*-regulating transcription factors modulates RAS transformed phenotypes including cellular viability, anchorage-independent growth and invasion-migration of pancreatic cancer cells. *RREB1* knockdown activates the amplitude and duration of RHOA via increased *ARHGEF2* expression. By relieving the negative regulation of RREB1 on the *ARHGEF2* promoter, we determined that ETS1 and SP3 are essential for the normal expression of *ARHGEF2* and contribute to the migratory behavior of pancreatic cancer cells. Furthermore, enforced expression of ARHGEF2 rescues loss of *SP3* driven invasion-migration and anchorage-independent growth defective phenotypes through restored activation of RHOA. Collectively, our results identify a transcription factor program required for RAS transformation and provide mechanistic insight into the highly metastatic behavior of pancreatic cancer.

## INTRODUCTION

Pancreatic cancer remains a lethal malignancy with a dismal five year survival rate of less than five percent. Pancreatic cancer is a highly metastatic disease presenting with metastasis to lymph nodes, liver and other distal sites [1]. Activating mutations in the KRAS proto-oncogene are nearly ubiquitous in pancreatic ductal adenocarcinoma (PDAC) and are found in 90-95% of cases [2, 3]. RAS genes activate signaling pathways that regulate transcription, proliferation, cell survival and motility all of which are commonly disturbed in cancer [4]. Oncogenic RAS activation engages diverse signaling pathways including RAF, PI3K, RAL-GDS and TIAM-1 that underlie the phenotypes associated with pancreatic cancer.

The RAS homology family (RHO-GTPases) function as molecular switches that cycle between a GTP bound active state and GDP bound inactive state. RHO family members include RHOA, B, and C as well as multiple RAC and CDC42 isoforms [5]. RHO proteins are activated by RHO guanine nucleotide exchange factors (GEFs) which catalyze the intrinsically slow nucleotide exchange from GDP to GTP and inactivated by RHO GTPase activating proteins (GAPs) which accelerate GTP hydrolysis. At the interface of multiple signaling pathways, RHO GTPases regulate the cellular cytoskeleton, morphology, cellular migration, cell survival and proliferation [5, 6, 7]. Activation of RHOA is required for transformation by oncogenic RAS through sustained MAPK signaling to promote proliferation and migration [7, 8, 9].

Previously, the RHO guanine exchange factor ARHGEF2 (also known as GEF-H1) was found to contribute to cell survival and growth in RAS-transformed cells [10]. ARHGEF2 is a microtubule associated Dbl family member of guanine exchange factors with specific exchange activity for RHOA [11]. The oncogenic potential of ARHGEF2 has been demonstrated in NIH-3T3 fibroblast transformation assays [12] and in nude mice [13]. *ARHGEF2* is amplified in hepatocellular carcinoma and promotes cell motility via activation of RHOA signaling [14]. The *ARHGEF2* gene is a transcriptional target of gain-of-function mutants of p53 [15] and the metastasis-associated gene hPTTG1 [16]. *ARHGEF2* was one of multiple genes that displayed an altered response after treatment with Imatinib in gastrointestinal stromal tumors [17] suggesting that targeting *ARHGEF2* expression could be an attractive therapeutic treatment for susceptible cancer types.

ARHGEF2 has been implicated in a myriad of cellular functions including roles in epithelial barrier permeability, cell motility and polarization, dendritic spine morphology, cell cycle regulation, and cancer (for review of ARHGEF2 function see [18]). ARHGEF2 plays a critical functional role in supporting RAS transformation as depletion of *ARHGEF2* hinders the growth of pancreatic xenografts *in vivo* [10]. ARHGEF2 protein levels were found to correlate with tumor progression in pancreatic tumor specimens and are acutely elevated by inducible expression of RAS [10]. However, the mechanism of transcriptional regulation of *ARHGEF2* downstream of RAS is unknown. In the present study, we identified a minimal RAS responsive promoter that drives *ARHGEF2* expression in a KRAS dependent manner downstream of multiple RAS signaling pathways. Importantly, we have identified a set of transcription factors required for transactivation of *ARHGEF2* by oncogenic KRAS that mediate survival, tumorigenicity and invasion-migration through *ARHGEF2* expression.

## RESULTS

### *ARHGEF2* is a transcriptional target of oncogenic KRAS and RAS signaling pathways

**Figure 1:**
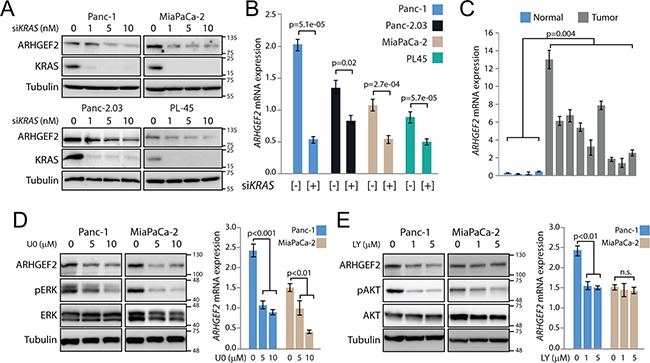
*ARHGEF2* is a transcriptional target of oncogenic KRAS **A**. Western blot analysis of ARHGEF2 in the indicated PDAC cell lines following acute knockdown of *KRAS* with the increasing concentrations of siRNA. Lysates were probed with indicted antibodies 72 hours post transfection. For this and subsequent experiments, tubulin served as a protein loading control. **B**. QPCR analysis of *ARHGEF2* mRNA in the indicated cell lines treated with control siRNA or siRNA targeting *KRAS* (10nM final concentration). Error bars in this and subsequent qPCR experiments represent standard deviations from three independent experiments. **C**. QPCR analysis of *ARHGEF2* mRNA expression in normal pancreas (Normal) and patient-derived xenografts (Tumor). The p-value represents significance between the normal and tumor group. **D**. Western blot and QPCR analysis of *ARHGEF2* in Panc-1 and MiaPaCa-2 cell lines treated with the indicated concentration of U0126 (U0). Lysates were probed with indicted antibodies 48 hours post treatment. **E**. Western blot and QPCR analysis of *ARHGEF2* in Panc-1 and MiaPaCa-2 cell lines treated with the indicated concentration of LY294002 (LY). Lysates were probed with indicted antibodies 48 hours post treatment.

Previously, we found ARHGEF2 protein levels were increased in cells transformed by each mutant RAS family member [10]. Since activating mutations of KRAS are found in over 90% of pancreatic cancers, we conjectured that oncogenic KRAS signaling would primarily regulate *ARHGEF2* expression in PDAC cells. Transient knockdown of *KRAS* with siRNA (Figures 1A, Supplementary Figure S1A and S1B) resulted in decreased *ARHGEF2* expression at the protein and mRNA levels in multiple PDAC cell lines harboring KRAS mutation under the same equimolar concentrations of siRNA (Figure 1A and 1B). To confirm that *ARHGEF2* expression was linked to KRAS expression, we examined *ARHGEF2* expression in human pancreatic ductal epithelial cells (HPDE) and HPDE cells transformed by oncogenic KRAS^G12D^ (HPDE-KRAS, Ref [19]). HPDE-KRAS cells exhibited increased expression of *ARHGEF2* mRNA and protein relative to non-transformed HPDE (Supplementary Figures S1C and S1D). Similarly, *ARHGEF2* expression was upregulated in NIH-3T3-KRAS^G12D^ compared to NIH-3T3 (Supplementary Figures S1C and S1D). In addition, *ARHGEF2* demonstrated significantly increased expression in nine patient-derived xenografts compared to normal pancreas tissue (Figure 1C).

Oncogenic RAS proteins engage multiple effector pathways required for transformation including the RAF/MEK/ERK pathway (MAPK) and the PI3K/AKT pathways [20]. To determine if *ARHGEF2* expression was a consequence of MAPK and/or PI3K activation, Panc-1 and MiaPaCa-2 cells were treated with the MEK1/2 inhibitor UO126 or the PI3K inhibitor LY294002. Levels of protein and *ARHGEF2* mRNA decreased following MEK1/2 inhibition in both cell lines (Figure 1D). *ARHGEF2* expression decreased following PI3K inhibition in Panc-1 cells but no change was observed with PI3K inhibition in MiaPaCa-2 (Figure 1E). These results show that *ARHGEF2* exhibits moderate to high expression in pancreatic tumors and is a transcriptional target of KRAS signaling principally downstream of MEK.

### A minimal RAS responsive promoter regulates *ARHGEF2*

The human *ARHGEF2* gene spans over 30 kb on chromosome 1 (Supplementary Figure S2A). Based on annotated NCBI reference sequence (RefSeq) transcripts for *ARHGEF2*, the transcription start site (TSS) was predicted to occur at either distal or proximal promoters (Supplementary Figure S2A). We cloned several DNA fragments around the distal promoter into a pGL3-promoterless luciferase reporter vector and were not able to drive luciferase expression of any construct tested in Panc-1 cells (data not shown). Since *ARHGEF2* is highly expressed in Panc-1 cells, we concluded that the proximal promoter was likely responsible for driving *ARHGEF2* expression and this region became the focus of further study.

**Figure 2:**
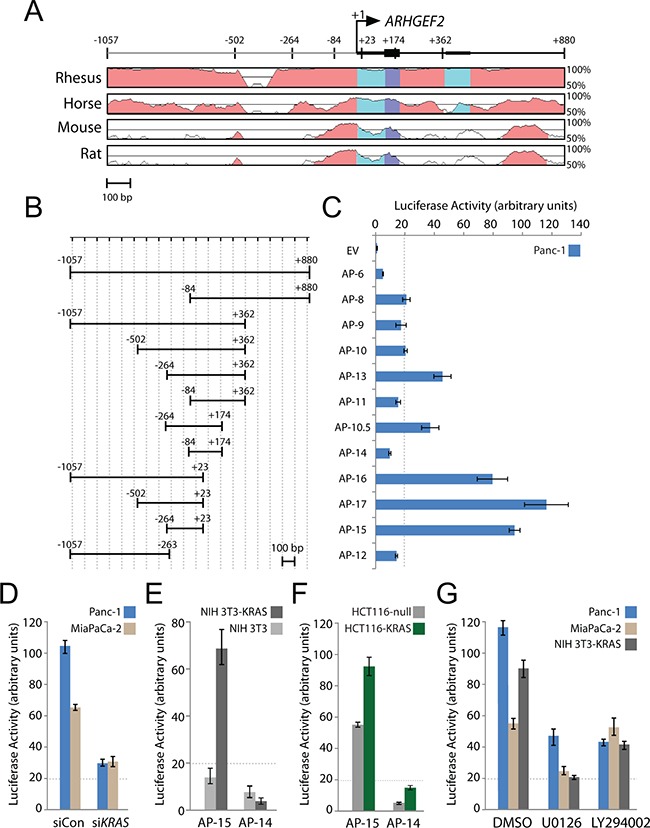
A highly conserved minimal promoter at the *ARHGEF2* transcription start site drives luciferase expression **A**. Phylogenetic conservation of the genomic region around the transcription start site (TSS) of *ARHGEF2*. VISTA (http://genome.lbl.gov/vista/index.shtml) was used to generate pairwise alignments between the human *ARHGEF2* sequence and homologous sequences in the indicated species. Graphs illustrate nucleotide identity for a 100 bp sliding window centered at a given position. Position +1 of *ARHGEF2* mRNA and the positions of the boundaries of promoter constructs used in panel B are mapped. **B**. Boundaries of *ARHGEF2* promoter constructs cloned into the pGL3-basic luciferase reporter vector. Numbers are relative to the TSS. Scale bar is 100 base pairs (bp). **C**. Normalized luciferase activity generated from the indicated *ARHGEF2* promoter (AP) construct transfected in Panc-1 cells. Luciferase activity was normalized to renilla expression and data is plotted as the fold change over cells expressing pGL3-promoterless empty vector (EV). Error bars in this and subsequent experiments represent standard deviations from three independent transfections. Luciferase expression below the dotted line was considered insignificant. **D**. Normalized luciferase activity from AP-15 promoter construct in a Panc-1 and MiaPaCa-2 cells pretreated with control siRNA (siCon) or siRNA targeting *KRAS* (si*KRAS*). **E**. Normalized luciferase activity from AP-15 and AP-14 promoter constructs in isogenic NIH-3T3 and NIH-3T3-KRAS cell lines. **F**. Normalized luciferase activity from AP-15 and AP-14 ARHGEF2 promoter constructs in isogenic HCT116-null and HCT116-KRAS cell lines. **G**. Normalized luciferase activity from AP-15 promoter construct in the indicated cell lines pretreated with DMSO, 10μM U0126 or 5μM LY294002.

The *ARHGEF2* proximal promoter contains regions of strong conservation suggesting an evolutionarily conserved promoter (Figure 2A) and contains high histone H3-Lysine27 acetylation (H3K27Ac) a mark indicative of actively transcribed chromatin supporting this region as the active promoter (Supplementary Figure S2A). A series of genomic fragments around the proximal *ARHGEF2* promoter (AP) were cloned into the pGL3-promoterless luciferase reporter plasmid (Figure 2B), many of which produced robust luciferase activity indicating functional promoters (Figure 2C). A minimal promoter (AP-15) containing the conserved region between -264 to +23 was the smallest fragment with the most robust activity (Figure 2C) and was functional in multiple PDAC cell lines (Supplementary Figure S2B). AP-14 was identified as the smallest fragment without activity and used as a control in subsequent experiments. Importantly, AP-15 promoter activity was diminished when both Panc-1 and MiaPaCa-2 cells were treated with siRNA targeting *KRAS* (Figure 2D) while AP-15 promoter was potently activated in NIH-3T3-KRAS^G12D^ relative to NIH-3T3 cells (Figure 2E). Increased luciferase expressed from the AP-15 reporter was also detected in isogenic HCT116-KRAS^G13D^ cells which express oncogenic *KRAS* relative to HCT116-null in which oncogenic *KRAS* has been knocked out by homologous recombination ([21]; Figure 2F). These data show that *ARHGEF2* is transactivated through a minimal KRAS-responsive promoter.

We next examined the effect of inhibiting RAS signaling pathways on AP-15 luciferase expression. AP-15 promoter activity was significantly reduced in multiple cell lines treated with the MEK1/2 inhibitor U0126 (Figure 2G). AP-15 promoter activity was diminished in PI3K inhibited Panc-1 and NIH-3T3-KRAS cells; however, no effect of the PI3K inhibitor on AP-15 activity was observed in MiaPaCa-2 after treatment with LY294002 (Figure 2G). We also examined the effect of a panel of small molecular inhibitors on AP-15 activity in Panc-1 cells. We analyzed a set of small molecule inhibitors targeting a variety of pathways and found that KRAS^G12D^ induction of AP-15 was suppressed by targeting binary combinations of the following signaling proteins: MEK plus RAC, MEK plus PI3K, JNK plus p38, RAC plus p38, or RAC plus PI3K. In distinction, AP-15 was activated by inhibition of either p38 or RHO (Supplementary Figure S2C, Supplementary Table S1). These results suggest that multiple signaling pathways impinge upon the *ARHGEF2* promoter in addition to the MAPK pathway.

### The *ARHGEF2* promoter is regulated by multiple transcription factors

**Figure 3:**
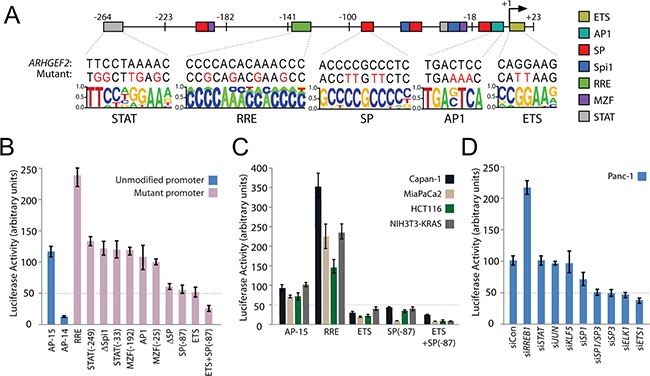
Identification and validation of transcription factors regulating the minimal *ARHGEF2* promoter **A**. Summary of putative transcription factor (TF) binding elements predicted in the AP-15 sequence. Locations of indicated TF binding sites are mapped to their respective location in AP-15 (colored boxes) and numbered relative to the *ARHGEF2* TSS (+1). The sequence of each element found in the endogenous promoter (*ARHGEF2*) and the bases mutated in the AP-15 mutant promoters (Mutant) are shown. **B**. Normalized luciferase activity generated from AP-15 and AP-14 promoter constructs (blue bars) or AP-15 promoters containing mutations in the indicated TF binding elements (pink bars) transfected in Panc-1 cells. Numbers in parenthesis refer to location of specific TF elements. **C**. Normalized luciferase activity generated from AP-15 promoter constructs or AP-15 promoters containing a mutant of the indicated TF binding element expressed in the indicated cell lines. **D**. Normalized luciferase activity generated from the AP-15 promoter construct in Panc-1 cells transfected with control siRNA (siCon) or siRNA targeting the indicated TFs.

To discover the transcription factors (TFs) that regulate *ARHGEF2*, an in-silico analysis of the AP-15 promoter sequence was performed using five prediction search algorithms (Supplementary Figure S3A). To increase our confidence in the predictions, only TF binding elements predicted by at least three search algorithms were considered. We found the AP-15 promoter was predicted to contain an ETS element (a binding site for ELK1 and ETS1, members of the ETS domain-containing family of transcription factors), an AP1 element (binding site for Fos-Jun), a MZF element (binding site for myeloid zinc factor-1), four GC boxes (binding elements for the specificity proteins SP1, SP3 and SP4 as well as KLF5), two Spi1 elements (ETS family member), a RAS responsive element (RRE, a binding site for the RAS responsive element binding protein RREB1), and a STAT response element (binding site for STAT1/3) (Figures 3A, Supplementary Figure S3A and S3B). Examination of RNAseq expression data averaged from twenty PDAC cell lines revealed that with the exception of MZF1, SP4, and Spi1 all TFs predicted to bind elements in AP-15 were expressed in the majority of PDAC cell lines (Supplementary Figure S3C).

To validate each TF binding element, we introduced mutations in AP-15 to disrupt TF binding as predicted by the position weight matrix (Figures 3A and Supplementary Figure S3B). Mutations introduced within the ETS element, all four SP elements (ΔSP) or the most highly conserved of the SP elements (SP^−87^) abrogated luciferase expression from the mutant promoter in Panc-1 cells and several other PDAC cell lines (Figure 3B and 3C). AP-15 with an ETS and SP^−87^ double mutation impaired promoter activity in all cell lines tested (Figure 3B and 3C). In contrast, mutations within the RRE augmented AP-15 driven luciferase expression in Panc-1 and several other PDAC cell lines (Figure 3B and 3C). Mutations introduced within AP1, MZF1, Spi1 and STAT binding elements had no effect on AP-15 dependent luciferase expression in Panc-1 cells (Figure 3B).

Since mutation of a TF binding element does not necessarily definitively disrupt binding of the TF with its promoter, we complemented the mutation studies with siRNA directed knockdown of each TF followed by analysis of AP-15 promoter activity. Efficient RNAi knockdown of each TF was confirmed by quantitative PCR (Supplementary Figure S3D). AP-15 promoter activity was diminished in Panc-1 cells treated with siRNAs targeting *ELK1*, *ETS1*, *SP1* or *SP3* (Figure 3D). Knockdown of *RREB1* increased expression of luciferase from AP-15 (Figure 3D). Knockdown of *JUN*, *KLF5*, and *STAT1/3* had no effect on promoter activity.

**Figure 4:**
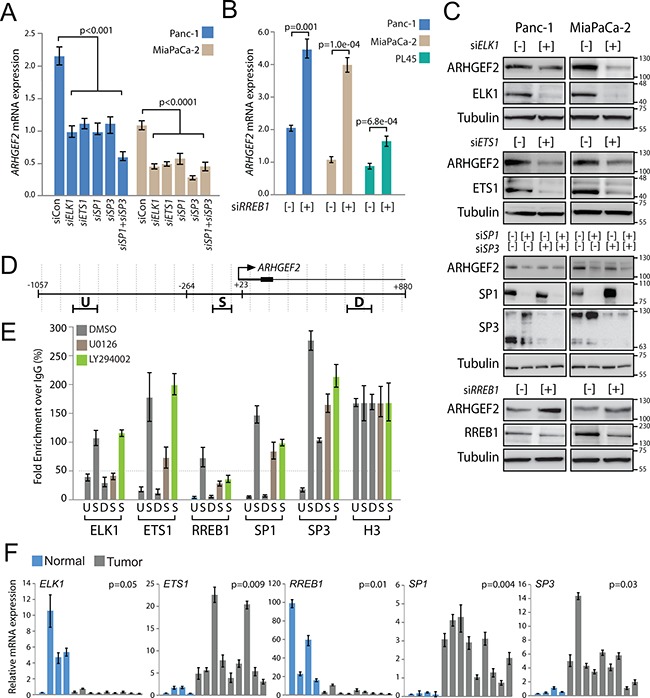
Validation of endogenous *ARHGEF2* transcriptional regulation **A**. QPCR analysis of *ARHGEF2* expression in Panc-1 and MiaPaCa-2 cells 72 hours post transfection with control siRNA or siRNA against the indicated TFs. **B**. QPCR analysis of *ARHGEF2* expression in the indicated PDAC cell lines 72 hours post transfection with control siRNA (−) or siRNA targeting RREB1 (+). **C**. Western blot analysis of ARHGEF2 in Panc-1 and MiaPaCa-2 cells 72 hours post transfection with control siRNA (−) or siRNA against the indicated transcription factors (+). Lysates were probed with indicted antibodies 48 hours post treatment. **D**. QPCR amplicons for ChIP were designed within 100-bp windows at the TSS (S), upstream of amplicon S (U), or downstream of amplicon S (D). The position of the highly conserved minimal promoter (−264 to +23) sequence is mapped relative to the TSS and the ChIP amplicons. **E**. QPCR analysis of chromatin immunoprecipitates from Panc-1 cells treated with DMSO, MEK inhibitor (U0126) or PI3K inhibitor (LY294002). Fold enrichment is calculated as the signal obtained after immunoprecipitation with the indicated antibody over IgG antibody and normalized to the signal obtained for histone H3 ChIP. The 50-fold enrichment threshold for positive transcription factor binding is indicated with a dashed line. Data are mean ± SEM from three independent measurements. **F**. QPCR analysis of the indicated TF expression in normal pancreas (Normal) and patient-derived xenografts (Tumor). The p value represents significance between normal and tumor group.

To validate endogenous *ARHGEF2* regulation, we examined *ARHGEF2* mRNA and protein levels in multiple PDAC cells treated with siRNA against each of the identified *ARHGEF2*-regulating TFs (Supplementary Figure S4A). Panc-1 and MiaPaCa-2 cells treated with siRNA targeting *ELK1*, *ETS1*, *SP1* or *SP3* individually had decreased *ARHGEF2* mRNA expression compared siRNA control treated cells by approximately fifty percent (Figure 4A). A third PDAC cell line, PL45, had decreased *ARHGEF2* mRNA expression with siRNA targeting *ELK1*, *ETS1*, and *SP3* but not *SP1* compared to siRNA control treated cells (Supplementary Figure S4B). All PDAC cell lines treated with siRNA targeting *RREB1* had significantly increased *ARHGEF2* expression (Figures 4B). In addition, expression of ARHGEF2 protein levels correlated to changes observed in *ARHGEF2* mRNA expression in lysates from siRNA treated cells (Figures 4C and Supplementary Figure S4C).

Lastly, chromatin immunoprecipitation (ChIP) was performed in Panc-1 cells to validate regulation of the endogenous *ARHGEF2* promoter. Amplicon windows for ChIP were designed at the TSS (S) and approximately 500 bases upstream and downstream (U and D) (Figure 4D). Since *ARHGEF2* is activated downstream of the MAPK and PI3K pathways in Panc-1 cells, ChIP was also performed in lysates obtained from Panc-1 cells treated with MEK1/2 inhibitor (U0126) or PI3K inhibitor (LY294002). Strong enrichment of the TSS amplicon (S) was observed in ELK1 and ETS1 precipitates from DMSO treated lysates which was decreased in MEK1/2 inhibited lysates (Figure 4E). No effect on ELK1 and ETS1 binding was observed in PI3K treated cells. Strong enrichment of the TSS amplicon (S) was observed in RREB1, SP1, and SP3 precipitates from DMSO treated lysates which was decreased by MEK1/2 or PI3K inhibition (Figure 4E). These results demonstrate that the endogenous *ARHGEF2* promoter is regulated by ELK1 and ETS1 in a MAPK dependent manner and regulated by RREB1, SP1 and SP3 through both the MAPK and PI3K pathways.

Collectively, our results demonstrate the *ARHGEF2* promoter is positively regulated by ELK1, ETS1, SP1 and SP3 and negatively regulated by RREB1. Importantly, all five of these transcription factors are upregulated in HPDE and NIH-3T3 cells transformed by oncogenic KRAS (Supplementary Figure S4D). We next interrogated tumors for dysregulated expression of *ARHGEF2*-regulating TFs. Using a panel of patient-derived xenografts and normal pancreas tissue, we observed elevated expression of *ETS1*, *SP1* and *SP3* and decreased expression of *ELK1* and *RREB1* in xenografts compared to normal pancreatic tissue (Figure 4F). These data suggest that the principle physiologic drivers of *ARHGEF2* transcription in pancreatic tumors are ETS1, SP1 and SP3.

### Transcriptional regulation of *ARHGEF2* mediates RAS-transformed phenotypes in pancreatic cancer cell lines

**Figure 5:**
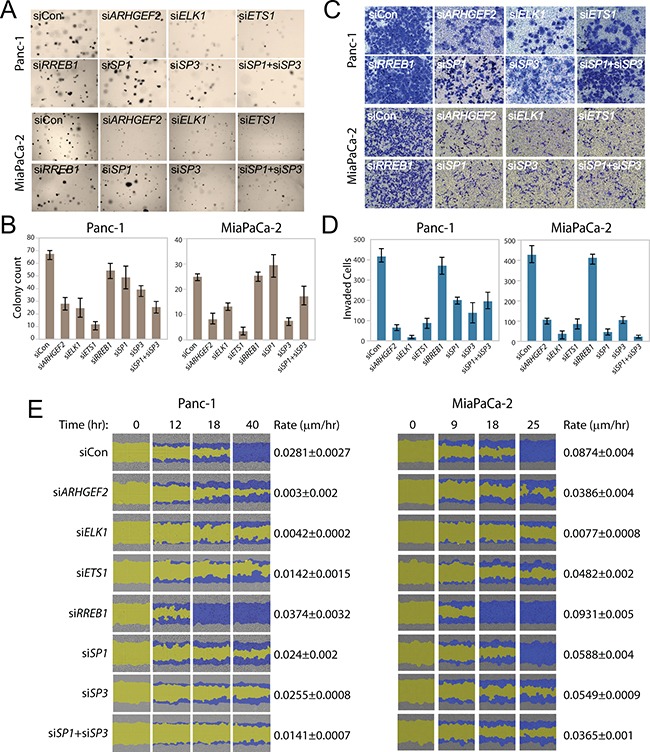
Transcription factors regulating *ARHGEF2* mediate RAS transformed phenotypes **A, B**. Representative images (A) and quantification (B) of Panc-1 and MiaPaCa-2 cells transfected with the indicated siRNA and grown for 7 days in 0.3% agar to form colonies. Bar graphs average colony counts from three representative images. **C, D**. Representative images (C) and quantification (D) of invasion assays in Panc-1 and MiaPaCa-2 cells through matrigel coated transwells following transfection with the indicated siRNAs. Bar graphs indicate the average number of invasive cells from three representative images. **E**. Scratch wound assay of Panc-1 and MiaPaCa-2 cells transfected with indicated siRNA and monitored over the time course. Images from Essen IncuCyte ZOOM. The initial scratch wound mask is colored yellow and the progression of cell migration is marked blue. Rate constants were derived by fitting wound closure curves to a first order rate equation (μm/hr).

We hypothesized that the *ARHGEF2*-regulating TFs could affect transformed phenotypes of PDAC cells in a manner correlating with loss or gain of *ARHGEF2* expression. We validated and used siRNA targeting *ARHGEF2* as a positive control for loss of *ARHGEF2* expression on effecting RAS-transformed phenotypes (Supplementary Figures S5A and S5B). We assessed the ability of Panc-1 and MiaPaCa-2 to support anchorage-independent growth following knockdown of *ARHGEF2*-regulating TFs. Transient depletion of *ELK1*, *ETS1, SP3* or the combined *SP1/SP3* knockdown suppressed colony formation by 50% or greater compared with siRNA control treated cells in both Panc-1 and MiaPaCa-2 similar to siRNA targeting *ARHGEF2* directly (Figure 5A and 5B). Knockdown of *RREB1* or *SP1* individually had no effect on colony formation in either cell line (Figure 5A and 5B). Importantly, we measured metabolic activity using AlamarBlue as quantitative measure of cell viability and proliferation following siRNA mediated knockdown of *ARHGEF2*-regulating TFs (Supplementary Figure S5C). We found that the viability of MiaPaCa-2 cells were exquisitely sensitive to *ARHGEF2*, *ETS1*, *ELK1* and *SP1/SP3* knockdown compared to Panc-1 cells (Supplementary Figures S5C and S5D). This result suggests ETS1 and SP1/SP3 are essential for PDAC viability in a subset of tumors and correlates with the expression of these genes observed in PDAC xenografts (Figure 4F).

Since ARHGEF2 is an exchange factor and RHO proteins modulate migratory cellular phenotypes, we next assessed the ability of Panc-1 and MiaPaCa-2 to activate invasion and migration following knockdown of *ARHGEF2*-regulating TFs. Panc-1 and MiaPaCa-2 cells demonstrated decreased invasion through matrigel coated transwells and decreased wound closing ability when treated with siRNAs targeting *ELK1*, *ETS1*, *SP1* or *SP3* phenocopying *ARHGEF2* knockdown and consistent with loss of *ARHGEF2* expression compared to control siRNA treated cells (Figures 5C, 5D and 5E). Knockdown of *RREB1* had no effect on invasion but significantly increased the rate of migration compared to siRNA control treated cells (Figures 5C, 5D and 5E).

It is conceivable that *ARHGEF2*-regulating TFs would mediate cellular phenotypes including invasion-migration through regulation of dozens of genes including other GEFs. The human genome encodes 143 known GEFs and GAPs for RHO (Supplementary Table S2), dysregulation of any or all through TF knockdown could mediate cellular viability, colony formation and invasion-migration. Therefore, we performed an *in silico* analysis of promoters of all RHOGEFs/GAPs for putative ETS, RRE and SP elements to see how frequently these TF binding sites would be found in a large subset of promoters (Figure S5E). The putative promoter of a given gene was defined as the 2kb region upstream of the first exon of the RefSeq gene. The TFSearch algorithm, set at a conservative 85% confidence level for finding a TF binding element, was used to examine promoter sequences. The data (Supplementary Figure S5E) is represented on a scale where 100% confidence of prediction was assigned a value of 1.0 and below 84% was considered not predicted. Slight differences in the sequence motifs for ELK1 and ETS1 enables TFSearch to distinguish between these two highly similar sequences (Supplementary Figure S5F). Based on predicted regulation, each gene was assigned to a group: RREB1+ELK1 regulated (I), RREB1 but not ELK1 regulated (II), ELK1 but not RREB1 regulated (III), SP regulated (IV), or ETS1 regulated (V). Accordingly, all genes in group I are predicted to have similar regulation as *ARHGEF2* and the majority of which are expressed in PDAC cell lines (Supplementary Figure S5E). The ETS1 and SP binding sites were ubiquitous and found in nearly all the examined promoters. An ELK1 binding site was predicted in approximately 50% of the promoters and a RRE was predicted in 17% of the promoters. These results highlight the possibility that the observed RAS-transformed phenotypes could be attributed to transcriptional dysregulation of multiple RHOGEFs/GAPs.

### RREB1 knockdown rescues ELK1 and SP1 knockdown on migration and growth phenotypes through the transcriptional activation of *ARHGEF2*

**Figure 6:**
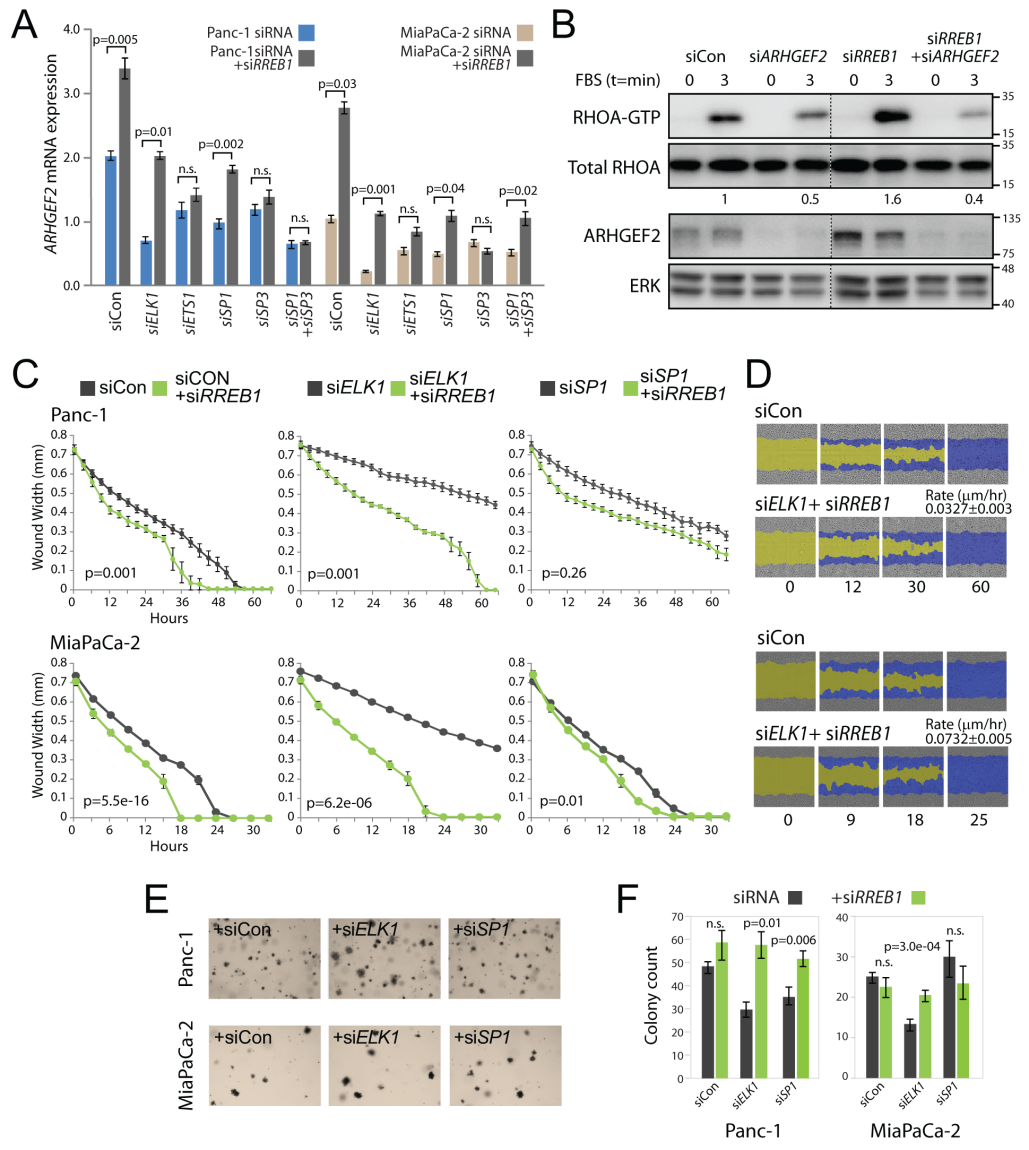
RREB1 knockdown restores *ARHGEF2* expression in the absence of ELK1 or SP1 to activate migration and colony formation **A**. QPCR analysis of *ARHGEF2* mRNA in Panc-1 and MiaPaCa-2 cells 72 hours post transfection with the indicated siRNA or siRNA plus si*RREB1*. P-values measure the statistic relevance of altered *ARHGEF2* expression between siRNA or siRNA plus si*RREB1*. For this and subsequent statistical analysis n.s. equals not significant. **B**. Western blot analysis of RHOA-GTP activation in Panc-1 cells transfected with the indicated siRNAs. 48 hours post siRNA transfection, serum starved cells were lysed (t=0 min) or stimulated with FBS (t=3 min) and then lysed. Lysates were incubated with Rhotekin-RHO binding domain protein beads, purified and subjected to western blot analysis. The ratio of RHOA-GTP/total RHOA is indicated. ERK served as a loading control. **C**. Wound width analysis of Panc-1 and MiaPaCa-2 cells transfected with the indicated siRNA (grey line) or siRNA plus si*RREB1* (green line) monitored over the time course. Graphs were generated with the Essen IncuCyte ZOOM. P-values calculated from wound width analysis at 40 hour time point (Panc-1) and 18 hour time point (MiaPaCa-2). **D**. Representative scratch wound images of Panc-1 and MiaPaCa-2 cells transfected with siCon (from Figure 5E) or siRNAs targeting *ELK1* plus *RREB1*. The initial scratch wound mask is colored yellow and the progression of cell migration is marked blue. The rate constants for wound closure for the combined knockdown of *ELK1* and *RREB1* are indicated (μm/hr). **E, F**. Representative images (E) and quantification (F) of Panc-1 and MiaPaCa-2 cells transfected with the indicated siRNA or the indicated siRNA plus siRNA targeting *RREB1* and grown for 7 days in 0.3% agar to form colonies. Bar graphs indicate the average colony counts from three independent experiments.

We have uncovered two regulatory elements within the *ARHGEF2* promoter, one specifying ELK1/ETS1 binding and the other specifying SP1/SP3 binding. We have also identified an RRE motif occupied by RREB1 that negatively regulates the *ARHGEF2* promoter. We hypothesized that combined knockdown of *RREB1* with the other *ARHGEF2*-regulating TFs would provide a hierarchical relationship of the most dominant TFs required for *ARHGEF2* expression. Panc-1 and MiaPaCa-2 cells were transfected with siRNA control or siRNA targeting *ELK1*, *ETS1*, *SP1* and/or *SP3* with or without siRNA targeting *RREB1*. In Panc-1 and MiaPaCa-2 cells, we found that the full de-repression of *ARHGEF2* expression was dependent on ELK1, ETS1, SP1 and SP3 (Figure 6A). However, combined siRNA targeting *ELK1* and *RREB1* or *SP1* and *RREB1* restored *ARHGEF2* mRNA expression to levels observed in siRNA control treated cells (Figure 6A), whereas the combined *ETS1* and *RREB1* knockdown or *SP3* and *RREB1* knockdown was unable to do so. These results demonstrate that ETS1 and SP3 are essential for activation of the *ARHGEF2* promoter and are dominant over the requirement for ELK1 and SP1.

We found that *RREB1* knockdown significantly enhanced the amplitude and duration of RHOA activation compared to siRNA control treated Panc-1 cells through increased *ARHGEF2* expression (Figure 6B and S6A). This suggested that relieving RREB1 repression of *ARHGEF2* in combination with knockdown of *ARHGEF2*-regulating TFs would determine if the observed RAS transformed phenotypes were dependent on expression of *ARHGEF2*. The combined knockdown of *RREB1* and *ELK1* was sufficient to restore the rate of diminished migration in the wound closing assay observed in the *ELK1* knockdown to that observed with cells treated with control siRNA (Figures 6C and 6D; compare to Figure 5E) suggesting an epistatic relationship between these two TFs. Combined *RREB1* and *SP1* knockdown moderately restored the migratory rate observed in *SP1* knockdown in Panc-1 and MiaPaCa-2 (Figure 6C). Knockdown of *RREB1* in combination with knockdown of *ETS1* or *SP3* was unable to restore the migratory rate observed when these TFs were knocked down alone (Supplementary Figure S6B). These results suggest that the deficiency in migration and invasion observed with *ELK1* and *SP1* knockdown was primarily through decreased expression of *ARHGEF2*.

Previously, ARHGEF2 has been shown to potentiate signaling through the MAPK pathway [10]. We hypothesized that increased *ARHGEF2* expression and activation of the MAPK pathway would rescue colony formation phenotypes seen with siRNA targeting *ARHGEF2*-regulating TFs. In Panc-1 and MiaPaCa-2 cells, the combined treatment of siRNA targeting *ELK1* and *RREB1* rescued the colony formation defect observed with *ELK1* knockdown alone (Figures 6E and 6F; compare to Figure 5A). Combined *SP1* and *RREB1* knockdown significantly increased colony formation over *SP1* knockdown alone in Panc-1 but no additional effect was observed in MiaPaCa-2 (Figures 6E and 6F; compare to Figure 5A). Knockdown of *RREB1* in combination with knockdown of *ETS1* or *SP3* was unable to alter colony formation ability over *ETS1* or *SP3* knockdown alone in either cell line (Supplementary Figures S6C and S6D).

### Enforced ARHGEF2 expression rescues defective invasion-migration and growth phenotypes associated with loss of SP3

**Figure 7:**
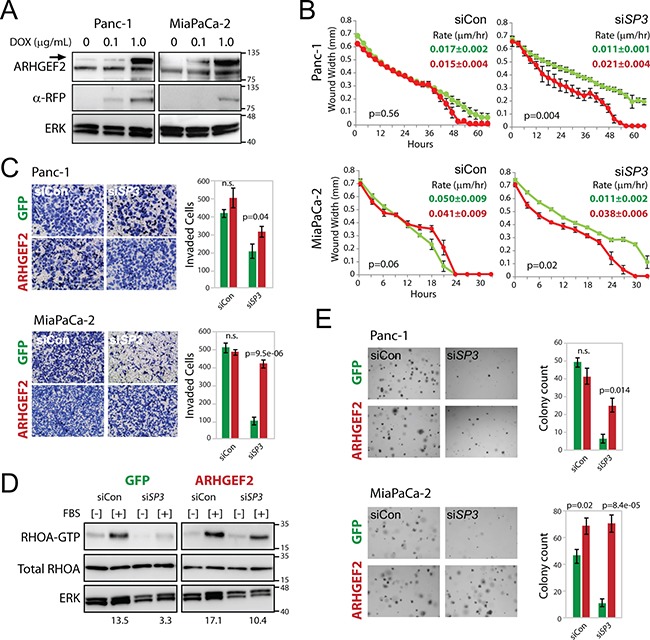
Enforced expression of ARHGEF2 rescues the *SP3* knockdown phenotypes through restored activation of RHOA **A**. Western blot analysis of doxycycline induced cherry-ARHGEF2 (upper band, arrow) in Panc-1 and MiaPaCa-2 cells treated with doxycycline (DOX). Lysates were collected 24 hours post treatment and probed with antibodies against ARHGEF2 or red fluorescent protein (RFP). ERK served as the loading control. **B**. Wound width analysis of GFP expressing (green lines) and cherry-ARHGEF2 expressing (red lines) Panc-1 and MiaPaCa-2 cells transfected with control siRNA (siCon) or siRNA targeting *SP3* (si*SP3*) monitored over the time course. Cells were grown in media supplemented with 0.1μg/mL doxycycline. Graphs were generated with the Essen IncuCyte ZOOM. P-values calculated from wound width analysis at 40 hour time point (Panc-1) and 18 hour time point (MiaPaCa-2). The rate constants for wound closure are indicated (μm/hr). **C**. Invasion of GFP and cherry-ARHGEF2 Panc-1 and MiaPaCa-2 cells through matrigel coated transwells following transfection with control siRNA (siCon) or siRNA targeting *SP3* (si*SP3*). Cells were grown in media supplemented with 0.1μg/mL doxycycline. Bar graphs indicate the average number of invasive cells from three independent experiments of GFP expressing cells (green bars) and cherry-ARHGEF2 expressing cells (red bars). **D**. Western blot analysis of RHOA-GTP expression in Panc-1 cells expressing GFP or cherry-ARHGEF2 and transfected with siRNA control (siCon) or siRNA targeting *SP3* (si*SP3*). Cells were grown in media supplemented with 0.1μg/mL doxycycline. 48 hours post siRNA transfection, lysates were collected following no stimulation [−] or 1 minute stimulation with FBS [+]. Lysates were incubated with Rhotekin-RHO binding domain protein beads, purified and subjected to western blot analysis. The ratio of RHOA-GTP/total RHOA is indicated. ERK served as the loading control. **E**. Representative images and quantification of GFP and cherry-ARHGEF2 Panc-1 and MiaPaCa-2 cells transfected with siRNA control (siCon) or siRNA targeting *SP3* (si*SP3*). Cells were grown in media supplemented with 0.1μg/mL doxycycline prior to replating for colony assay. Cells were grown for 7-9 days in 0.3% agar to form colonies. Bar graphs show the average colony counts from three representative images of GFP expressing cells (green bars) and cherry-ARHGEF2 expressing cells (red bars).

Since decreased migration and colony formation observed with *ETS1* and *SP3* knockdown could not be restored by removing RREB1 negative regulation of the *ARHGEF2* promoter, we wanted to determine if defects in these RAS transformed phenotypes could be attributed directly to decreased *ARHGEF2* expression. Using Panc-1 and MiaPaCa-2 cells, we created stable cell lines that contained a doxycycline inducible cherry-ARHGEF2 or a doxycycline inducible GFP as a control (Figures 7A and Supplementary Figure S7A). Inclusion of doxycycline in the growth media induced the expression of cherry-ARHGEF2 or GFP without effecting endogenous ARHGEF2 protein levels (Figures 7A, Supplementary Figure S7A and S7B).

We examined the phenotypes observed with knockdown of the *ARHGEF2*-regulating TFs in Panc-1 and MiaPaCa-2 with concurrent doxycycline induced *ARHGEF2* transgene expression. Enforced expression of cherry-ARHGEF2 rescued the rate of migration and invasion in *SP3* knockdown cells to those comparable to siRNA control treated cells in both Panc-1 and MiaPaCa-2 (Figure 7B and 7C). In the presence of *SP3* knockdown, Panc-1 cells expressing dox-induced GFP had decreased RHOA activation compared to siRNA control treated cells (Figure 7D). However, enforced cherry-ARHGEF2 expression was able to rescue RHOA activation in the presence of *SP3* knockdown (Figure 7D). A minor but significant increase in migration was observed in MiaPaCa-2 cells with enforced expression of cherry-ARHGEF2 in the presence of *ELK1* knockdown compared to siRNA control (Supplementary Figure S7C). However, doxycycline induced expression of cherry-ARHGEF2 was unable to rescue wound closure of Panc-1 cells treated with siRNA targeting *ELK1*, *ETS1* or *SP1* or MiaPaCa-2 cells treated with siRNA targeting *ETS1* or *SP1* (Supplementary Figure S7C). These results suggested that defective migration and invasion phenotypes with *SP3* knockdown were due to the loss of *ARHGEF2* expression and subsequent decreased activation of RHOA.

Additionally, the impact of enforced cherry-ARHGEF2 expression on colony formation was examined in cells treated with siRNAs against *ARHGEF2*-regulating TFs. Enforced expression of cherry-ARHGEF2 significantly enhanced colony formation in MiaPaCa-2 cells but had no additional effect on colony formation in Panc-1 cells treated with control siRNA (Figure 7E). Enforced cherry-ARHGEF2 expression rescued colony formation with knockdown of *SP3* in both Panc-1 and MiaPaCa-2 cell compared to GFP expressing cells (Figure 7E). However, the decrease in colony formation observed with knockdown of the other TFs was not rescuable in either cell line with dox-induced cherry-ARHGEF2 (Supplementary Figure S7D). These results reveal an essential role for SP3 in transactivation of the *ARHGEF2* promoter downstream of oncogenic KRAS and modulation of invasion-migration and anchorage independent growth of pancreatic cancer cells.

## DISCUSSION

**Figure 8:**
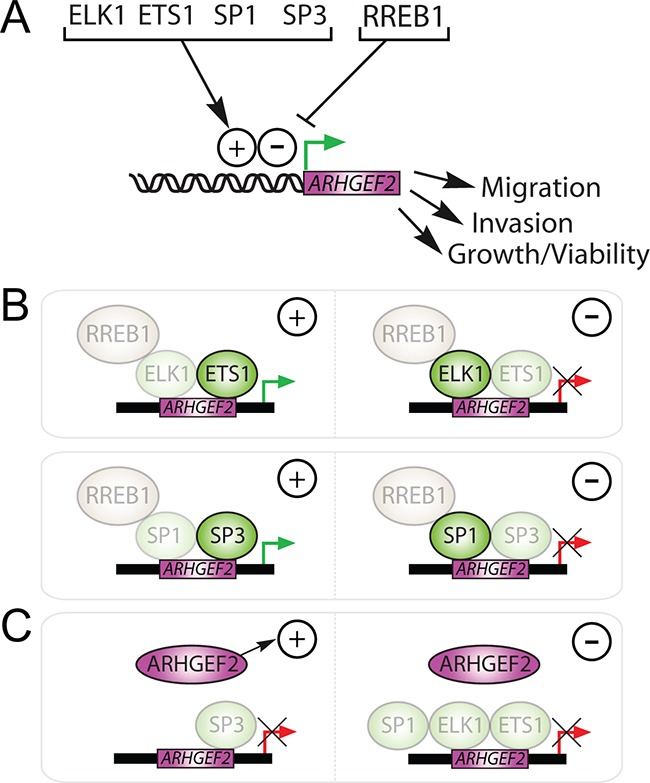
Summary of *ARHGEF2* transactivation and regulation of RAS mediated phenotypes via *ARHGEF2*-regulating TFs **A**. The transactivation of the *ARHGEF2* promoter by ELK1, ETS1, SP1, and SP3 activates RAS mediated phenotypes (+), whereas repression of *ARHGEF2* by RREB1 inhibits RAS mediated phenotypes (−). **B**. Activated *ARHGEF2* expression (green arrows) rescued RAS mediated phenotypes (+) observed with knockdown of *RREB1, ELK1, or SP1* (left); however, knockdown of *RREB1, ETS1, or SP3* (right) failed to activate *ARHGEF2* expression (red arrows) or rescue RAS mediated phenotypes (−). Shaded TFs represent knocked down genes. **C**. Enforced expression of ARHGEF2 rescued RAS mediated phenotypes (+) observed with knockdown of *SP3* (left) but could not rescue (−) phenotypes associated with knockdown of *ELK1, ETS1, or SP1* (right). Shaded TFs represent knocked down genes.

KRAS signaling modulates TF activity resulting in temporal and spatial changes in gene expression. We have discovered a set of TFs downstream of KRAS that converge to regulate the expression of *ARHGEF2* required for cell survival, anchorage independent growth, and invasion-migration. We have found that the *ARHGEF2* promoter is activated by ELK1, ETS1, SP1 and SP3 and repressed by RREB1 (Figure 8A). We have determined that transcriptional activation of *ARHGEF2* results in increased migration, invasion, and growth and repression of *ARHGEF2* decreased these phenotypes. The combined knockdown of *RREB1/ELK1* or *RREB1/SP1* permitted the induction of *ARHGEF2* expression and stimulated ARHGEF2 mediated phenotypes indicating that negative regulation by RREB1 is dominant over both ELK1 and SP1 (Figure 8B). Conversely, we found that the induction of *ARHGEF2* transcription and ARHGEF2 mediated phenotypes were not increased by the combined knockdown of *RREB1/ETS1* or *RREB1/SP3* (Figure 8B). These results demonstrate that the *ARHGEF2* promoter is dominantly regulated by ETS1 and SP3 which are essential for activation of the promoter. Furthermore, using over expression approaches we found that enforced expression of ARHGEF2 rescued SP3 deficient phenotypes but was not able to rescue defective phenotypes associated with knockdown of the other *ARHGEF2*-regulating TFs (Figure 8C). These data indicate that SP3 regulates invasion-migration and colony formation primarily through transactivation of *ARHGEF2* whereas ELK1, ETS1, and SP1 likely modulate these phenotypes through multiple genes including *ARHGEF2*.

RAS proteins engage diverse signaling pathways to regulate diverse cellular outcomes and tumorigenic phenotypes. We have found that *ARHGEF2* is transcriptionally activated by signaling through multiple RAS effector pathways including the MAPK and PI3K pathways. We have shown that MAPK and PI3K drive expression of *ARHGEF2* in Panc-1 and NIH-3T3 cells. Previously, HRAS-transformed fibroblasts, OV-90, HCT116, and Panc-02.03 cells were found to regulate ARHGEF2 protein levels through the MAPK pathway but not PI3K [10] suggesting that PI3K regulation of *ARHGEF2* is functional only in a subset of cells. Using small molecule inhibitors, we have established that activation of *ARHGEF2* expression is controlled through JNK, RAC, and TGF-β pathways while transcriptional inhibition of *ARHGEF2* is mediated through p38 and RHO. Previously, *ARHGEF2* has been found to be a target gene of TGF-β signaling and promote invasion and migration through activation of RHO [22, 23]. Our data suggests that RHO functions as part of a negative enzyme-product feedback loop to buffer the expression of *ARHGEF2*.

RHO has a demonstrated role in the RAS-transformation program [7, 8, 9]. Our results suggest RHO activation occurs downstream of the RAS-MAPK pathway mediated by ARHGEF2 and is essential for the enhanced migratory behavior of PDAC cells. The reinforced expression of *ARHGEF2* by oncogenic KRAS promotes positive feedback through the MAPK pathway and increased RHOA-GTP levels. However, our findings support the likelihood that oncogenic RAS drives metastasis through the transcriptional dysregulation of multiple RHOGEFs/GAPs. Our data suggest that the promoter regions of many RHOGEFs and RHOGAPs have similar potential transcriptional binding sites as *ARHGEF2* and that RAS may regulate a complex network of RHO-GTPases that contribute to tumor survival, proliferation and metastatic phenotypes. For example, RHOA activation by oncogenic RAS was previously found to depend on cytosolic p190-RHOGAP activity [9]. Future experiments will be necessary to explicate the transcriptional regulation of all the RHOGEFs and GAPs regulated downstream of oncogenic RAS.

Migratory cellular behavior is regulated through multiple signaling pathways in addition to RHO including the RAL GTPases that act as key effectors for Ras transformation. In MiaPaCa-2 cells, RHOA and RALA signaling pathways were found essential for KRAS dependent regulation of migration and invasion required for pancreatic cancer metastasis [24]. Divergent roles for the RAL proteins have been identified and suggest RALA is critical for tumor initiation whereas RALB is important for tumor metastasis in pancreatic cancer [25]. Interesting, RALB was required for invasive lung cancer cell behavior through a mechanism dependent on ARHGEF2 activation of the RHOA/ROCK pathway [26]. These results suggest multiple levels of cross-talk between the RAS, RHO, and RAL pathways for regulation of invasion and migration. Although the mechanism of invasive cellular behavior is not well understood, regulation of structures such as invadopodia comprised of an actin cores enriched in actin-binding proteins, adhesion molecules, integrins, and scaffold proteins for example, are known downstream targets of ARHGEF2-RHOA signaling and suggest ARHGEF2 acts as a RAS effector for the regulation of protein structures required for migratory cellular behavior.

We have found that *ARHGEF2* is transactivated by ELK1 and ETS1 downstream of the KRAS-MAPK pathway and demonstrate ETS1 is sufficient to activate *ARHGEF2* in the absence of ELK1. The ETS transcription factors, including ELK1 and ETS1 are a large family of transcription factors that include 28 genes in humans that play important roles in development, proliferation and transformation [27]. Gene regulation by the ETS transcription factors are mediated through diverse signaling pathways such as MAPK, p38, JNK, and PI3K consistent with regulatory input of the *ARHGEF2* promoter we have identified. A genome-wide expression analysis showed that ETS1 was required for activation of RAS-regulated cell migration in epithelial cells including Panc-1 [28]. ELK1 in addition to regulation of *ARHGEF2* activates invasion-migration through other RAS target genes including the micro-RNA miR-31 [29].

We have found that the SP1 and SP3 proteins are both required for *ARHGEF2* promoter activation but SP3 regulates invasion-migration and anchorage-independent growth explicitly through expression of *ARHGEF2* and downstream activation of RHOA. The specificity proteins are a large family of zinc finger transcription factors with over 16 members known to regulate GC-rich elements widely distributed in promoters, enhancers and locus control regions [30]. Both SP1 and SP3 are phosphorylated downstream of the MAPK pathway [31] and the interaction of SP1 and SP3 recruits the transcriptional initiation complex to TATA-less gene promoters [32]. The fact we were unable to detect a TATA-box in the *ARHGEF2* promoter is consistent with a role for SP1 and SP3 in activating *ARHGEF2* expression. Interestingly, the AP-15 and AP-14 promoter constructs differ mainly in the inclusion or exclusion respectively of the SP binding element, suggesting differences in their promoter activities is due to SP1 and/or SP3.

The RAS responsive element binding protein 1 (RREB1) is a zinc finger transcription factor initially identified as a target of the RAS/RAF signaling cascade and has been shown to both activate and repress transcription of genes in response to RAS pathway activity [33]. RREB1 has emerged from multiple screens as a likely human oncogene and a driver of colorectal cancer [34, 35]. However, the role of RREB1 is complex and can demonstrate both oncogenic and tumor suppressive properties in different systems. For example, RREB1 has been shown to negatively regulate the micro-RNA miR-143/145 promoter and shRNA knockdown of RREB1 in a normal pancreatic ductal cell line transformed by KRAS abolished growth in soft agar [36]. However, temporal expression of RREB1 may be important for tumor development and maintenance. For example, we observe that RREB1 influences the amplitude of RHOA activation and modulates ERK signaling through regulation of *ARHGEF2*. In addition, RREB1 regulates the *ELK1* promoter (Kent and Rottapel, unpublished results) suggesting a feedback mechanism for regulation of ELK1 targets through the MAPK pathway and provides another important mechanistic layer for ERK–negative feedback regulation of the MAPK pathway through the transcription factor RREB1. RREB1 expression has also been shown to decrease during PanIN development in pancreatic cancer [37] consistent with our results observed in tumor xenografts. Early down regulation of *RREB1* may promote RAS-mediated metastasis through increased *ARHGEF2* expression. This is consistent with previous observations that transformed pancreatic tumor cells can disseminate prior to tumor formation in a mouse model of pancreatic cancer [38].

In conclusion, we have uncovered a set of transcription factors that impact colony formation and invasion-migration of pancreatic cancer cells through their transcriptional regulation of *ARHGEF2*. Collectively, oncogenic KRAS promotes signal diversification through this set of TFs which transactivate *ARHGEF2* in addition to other target genes needed to mediate the RAS-transformation program.

## MATERIALS AND METHODS

### Cell culture

All cell lines used in the study (Jason Moffat, University of Toronto) were cultured as previously described [10]. Stable GFP and Cherry-ARHGEF2 inducible cells were established by co-transfection with lentivirus containing Tet-On3G-system (Clontech) and selecting transfected cells with 200ng/mL G418 and 1μg/mL puromycin (Sigma).

### Cell viability and colony formation

Viability assays were performed in 96-well plates with 8 replicates for each condition and analyzed with AlamarBlue (Thermofisher) according to the manufacturer's protocol. Colony formation assays were performed with 3 replicates for each condition. Briefly, 5000 cells/mL were suspended in warm 0.35% agarose in DMEM (4.5 mg/ml glucose) supplemented with 10% FBS in the absence of antibiotics and layered on a 0.5% agarose/DMEM base layer. Cells were grown for 7-10 days and then photographed.

### Chromatin immunoprecipitation

ChIP was performed in Panc-1 cells using SimpleChIP Enzymatic Chromatin IP kit with magnetic beads (Cell Signaling #9003) following the manufacturers’ protocol. Antibodies used for immunoprecipitation are listed in Supplementary Table S3. The Sigma-Aldrich RREB1 antibody was concentrated 10-fold using Amicon affinity concentrator (EMD Millipore) prior to ChIP. Primer sequences for ChiP amplicons are provided in Supplementary Table S4.

### Gene expression analysis

Cells were transfected with siRNA against *KRAS* (siGenome) or TFs (Silencer select, Invitrogen) at 1-5nM final concentration using RNAiMax (Invitrogen) according to the manufacturer's protocol. Total RNA was isolated from cells with TRIZOL (Invitrogen). Excess tissues from resected pancreatic carcinomas were implanted in nude mice to generate xenografts as described [39]. After harvesting, RNA was extracted from xenografts using the miR-VANA kit (Ambion) according to the manufacturer's protocol. cDNAs were made using the QuantiTect kit (Qiagen). All QPCR experiments were performed using Fast SYBR Green master mix (ThermoFisher) and transcript abundance was normalized to β-actin mRNA expression. QPCR was performed using an ABI7900 system with the Fast-SYBR Green PCR core reagent (Applied Biosystems). Primer sequences are provided in Supplementary Table S4. All QPCR experiments were repeated three times.

### Migration-invasion assays

For migration assays, cells were plated at confluency in a 96-well plate with 8 replicates for each condition. Scratches were made with the Essen Biosciences scratch wound maker. Wound closure was monitored using the Essen IncuCyte ZOOM system. Invasion assays were performed with 3 replicates for each condition. Transwells (Corning-3422, 8μm pore size) were coated with 1:40 dilution of cold matrigel in PBS, dried overnight, and rehydrated with serum free media for 2 hours prior to use. Cells were added to the upper chamber in base media and complete media containing 20% FBS was added to the lower chamber. Following 24-48 hours invasion, transwells were washed with PBS, stained with crystal violet/70% ethanol. Matrigel was removed carefully and membranes photographed under the light microscope.

### Molecular cloning

Primer sequences used for promoter cloning are provided in Supplementary Table S4. All promoter sequences were amplified from human genomic DNA (Roche) using Q5 high-fidelity 2X master mix (NEB). PCR products were gel purified and cloned into pGL3-Basic luciferase vector (Promega) utilizing the BglII and NheI restriction sites. Mutations in TF binding elements were introduced using the QuikChange Site-directed mutagenesis kit (Stratagene). The ΔSP1 construct was creating using Gene Art (Thermo Fisher Scientific).

### Promoter assays

Luciferase reporter assays were conducted using the Dual-Luciferase Reporter Assay System (Promega) and reading on a Glo-Max dual injector luminometer (Promega). Cells were transfected with 100 ng of pGL3-promoter reporter construct and 4 ng of phRL-SV40 (Promega) using Lipofectamine-2000 (Invitrogen). 18 hours post transfection cells were lysed and assayed for firefly and renilla luciferase activity. Where indicated siRNA (Invitrogen) were co-transfected at 5nM final concentration. For pathway inhibition experiments, serum starved cells were treated with small molecule compounds at concentrations indicated (Supplementary Table S1) in growth media two hours prior to reporter transfection. All luciferase experiments are an average of 3-6 independently repeated experiments.

### RHOA activation assay

Serum starved cells were stimulated with 10% FBS (final concentration) for 1 minute to activate RHO. RHOA-GTP pulldown was performed with RHOA activation kit (Cytoskeleton) following the manufacturer's protocol.

### Statistics and data analysis

Statistical analysis was done using Student's t-test, assuming equal variance, and p-values were calculated based on two-tailed test. Colony assay images and crystal violet stained transwell membranes were quantified using OpenCFU. First-order rate constants were calculated with GraFit7.0. In-silico transcription factor binding predictions were done with ConSite (http://consite.genereg.net), ConTraV2 [40], MATCH [41], TFSearch (www.cbrc.jp/research/db/TFSEARCH.html), and the USCS genome browser (Feb 2009 assembly).

### Western blot

Standard protocols were followed and blots were quantified with BIO-RAD Quantity One. All antibodies used are provided in Supplementary Table S3.

## SUPPLEMENTARY FIGURES AND TABLES




