# Supplementary material for: An oncogenic KRAS transcription program activates the RHOGEF ARHGEF2 to mediate transformed phenotypes in pancreatic cancer
Source: Oncotarget. 2016 Nov 7;8(3):4484–500. doi: 10.18632/oncotarget.13152 (PMC5354848; doi:10.18632/oncotarget.13152)
Supplement: Supplementary file 1 [file oncotarget-08-4484-s001.pdf]

An oncogenic KRAS transcription program activates the RHOGEF *ARHGEF2* to mediate transformed phenotypes in pancreatic cancer

SUPPLEMENTARY FIGURES AND TABLES

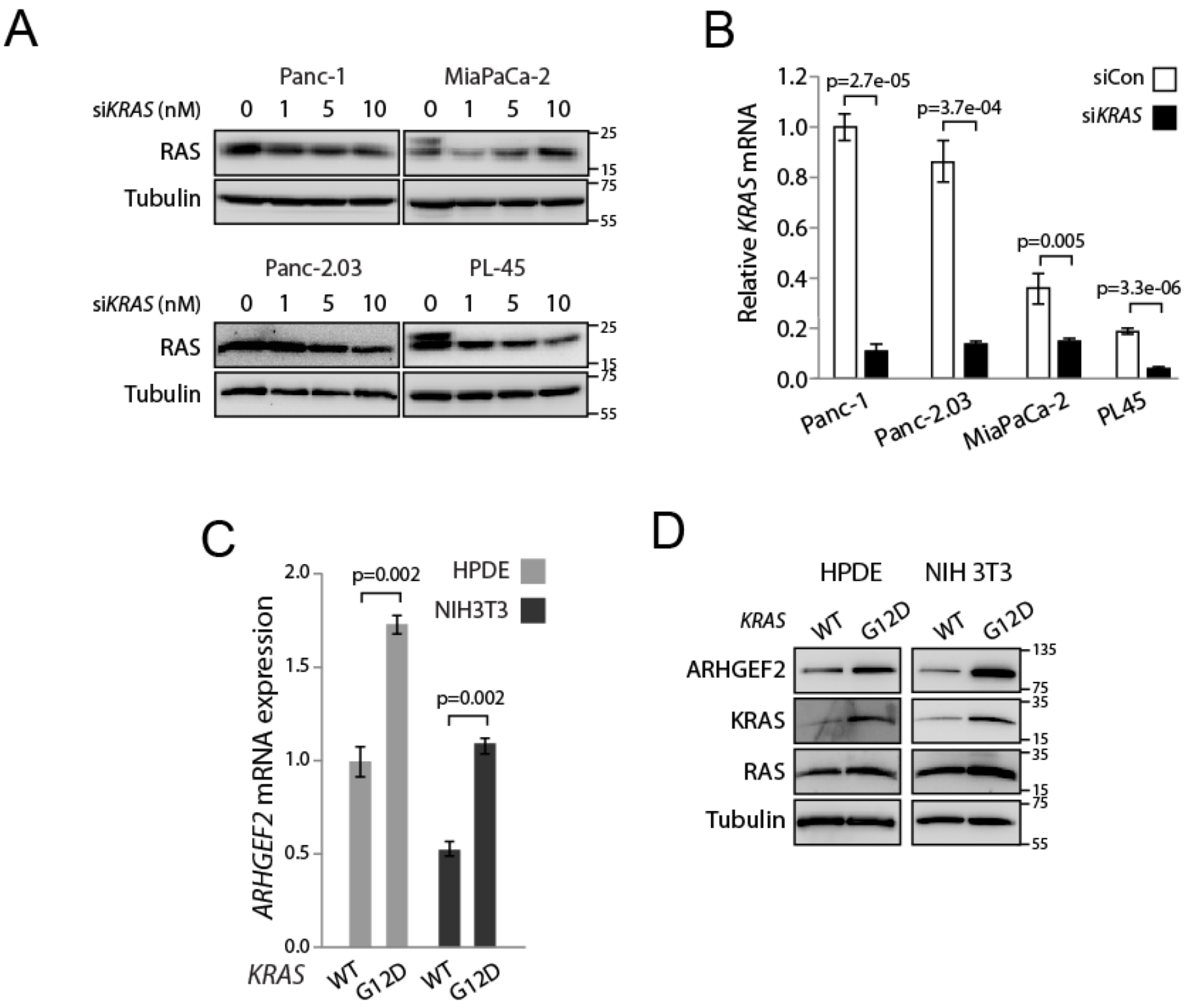

Supplementary Figure S1: A. Western blot analysis of total RAS in the indicated cell lines following acute knockdown of *KRAS*. Lysates were probed with indicted antibodies 72 hours post transfection. **B.** Quantitative PCR analysis of *KRAS* mRNA expression in the indicted cell lines treated with control siRNA (siCon) or siRNA targeting *KRAS* (si*KRAS*). **C, D.** Quantitative PCR analysis of *ARHGEF2* mRNA (C) and western blot analysis of ARHGEF2 protein (D) and in HPDE and NIH 3T3 cells with wild type *KRAS* (WT) and transformed with oncogenic KRAS^{G12D} (G12D).

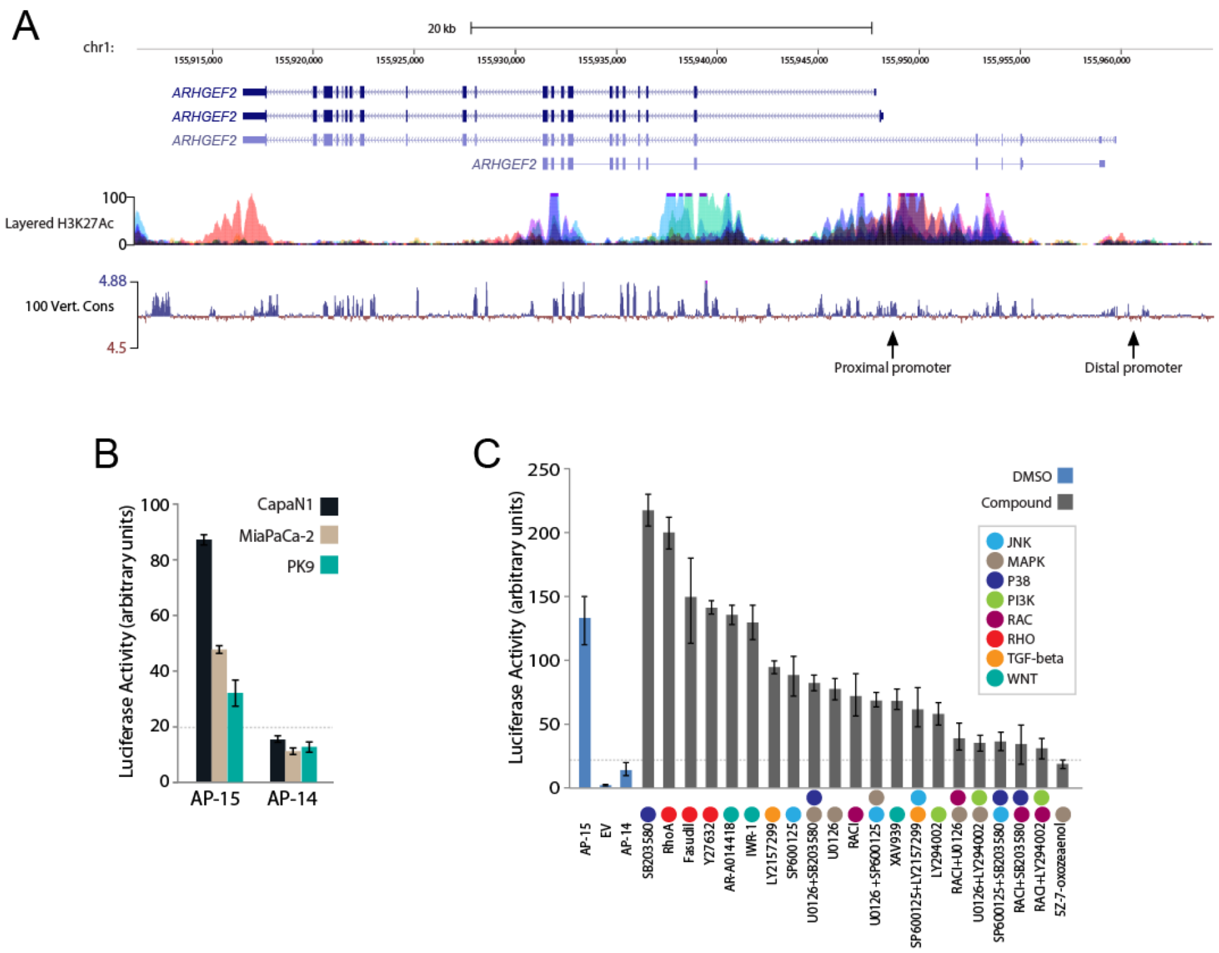

Supplementary Figure S2: A. Genomic region of chromosome 1 encompassing the human *ARHGEF2* gene. The plots depicted below the transcript show the layered H3K27Ac marks and the evolutionary conservation (UCSC Genome Browser 28 species conservation track, NCBI36/hg18 assembly). Location of the proximal promoter and an alternative distal promoter are referenced. **B.** Normalized luciferase activity from AP-15 and AP-14 *ARHGEF2* promoter construct in the indicated pancreatic cancer cell lines. **C.** Normalized luciferase activity generated from AP-15 and AP-14 promoter constructs (blue bars) or AP-15 promoter in cells pretreated with the indicated small molecule compounds (grey bars) transfected in Panc-1. Signaling pathways affected by the compounds are color coded. See Supplementary Table S1 for additional information on compounds.

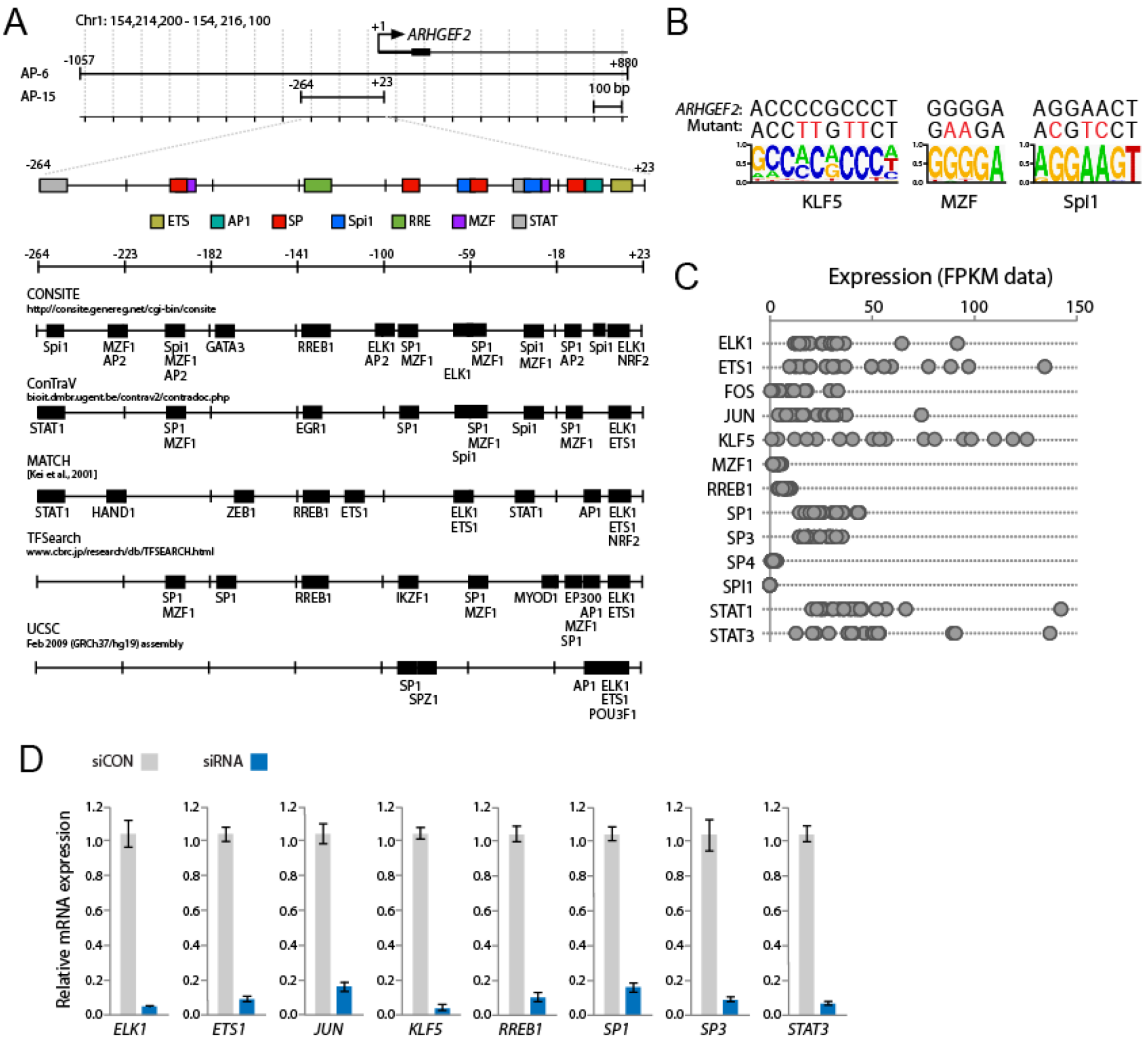

Supplementary Figure S3: A. Transcription factors predicted to bind AP-15 minimal *ARHGEF2* promoter sequence. (Top) The minimal -264 to +23 sequence is shown relative to the *ARHGEF2* TSS (+1) and the AP-6 sequence. (Bottom) The AP-15 sequence was examined using the indicated prediction software for TF binding elements. TF binding sites and their respective locations in AP-15 are annotated by solid black boxes. TF elements that were predicted 3 or more times are summarized in the expanded -264 to +23 sequence and annotated with color coded boxes. **B.** Position weight matrices for KLF5, MZF1, and Spi1 transcription factors. The KLF5 sequence is represented by the SP1/SP3 (red box) binding site in AP-15 due to overlap. **C.** FPKM expression data from 20 PDAC cell lines of all TFs represented in panel A. **D.** Quantitative PCR analysis indicated mRNAs of genes targeted by siRNAs used in this study.

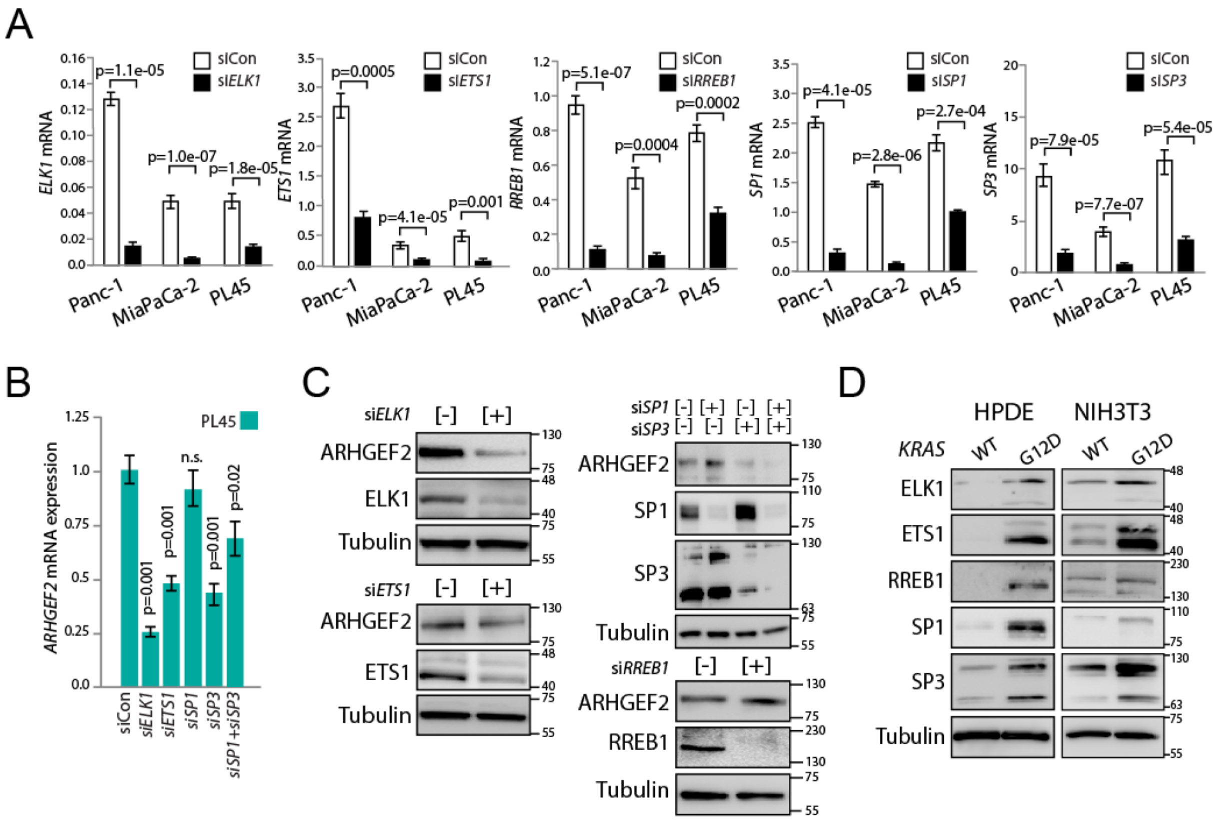

Supplementary Figure S4: A. Quantitative PCR analysis indicated TF mRNAs following siRNA directed knockdown in the indicated cell lines. Samples were used for validation of ARHGEF2 expression in Figures 4A and 4B. **B, C.** QPCR analysis of *ARHGEF2* mRNA (B) and western blot analysis of ARHGEF2 in PL45 cells 72 hours post transfection with control siRNA or siRNA against the indicated TFs. **D.** Western blot analysis of the indicated *ARHGEF2*-regulating TFs in lysates obtained from HPDE and NIH 3T3 cells with wild type *KRAS* (WT) and transformed with oncogenic $KRAS^{G12D}$ (G12D).

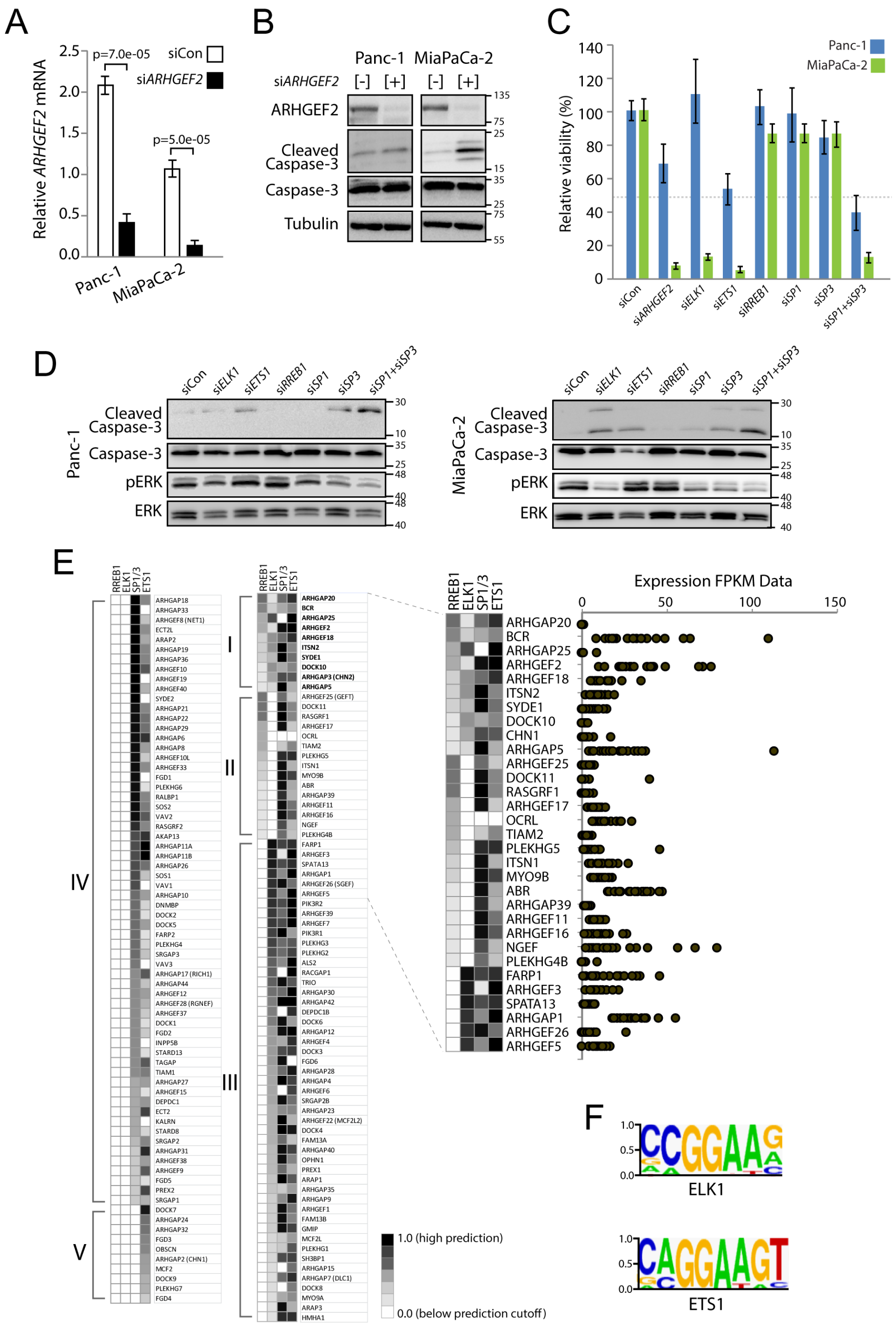

Supplementary Figure S5: A. Quantitative PCR analysis of ARHGEF2 mRNA in Panc-1 and MiaPaCa-2 cells treated with siRNA against *ARHGEF2*. **B.** Western blot analysis of cleaved caspase-3 in Panc-1 and MiaPaCa-2 cells treated with siRNA against *ARHGEF2*. **C.** Relative viability of Panc-1 and MiaPaCa-2 cells 72 hours post transfection with siRNA against the indicated *ARHGEF2*-regulating TFs. Cells were incubated with alamarblue for 2 hours prior to reading the fluorescence at 565nm. The 50% viability indicated with a dotted line. Error bars average two independent experiments with 8 replicates per condition each. **D.** Western blot analysis of cleaved caspase-3 and activated ERK in Panc-1 and MiaPaCa2 cells treated with siRNA against the indicated *ARHGEF2*-regulating TFs. Lysates were probed for caspase-3 cleavage 72 hours post transfection. **E.** Heat map analysis of predicted RREB1, ELK1, SP1, and ETS1 binding elements in the proximal promoters of all predicted RhoGAPs and RhoGEFs in the human genome. The heat map was subdivided into groups (Roman numerals I-V) based on the following predicted regulation: group I RREB1+ELK1 regulated; group II RREB1 but not ELK1 regulated; group III ELK1 but not RREB1 regulated; group IV SP1 regulated, group V ETS1 regulated. Expanded view of the group I, group II and top 5 of group III targets compared to their respective FPKM expression from 20 PDAC cell lines for each gene shown on the right. **F.** Position weight matrices for ELK1 and ETS1 transcription factors.

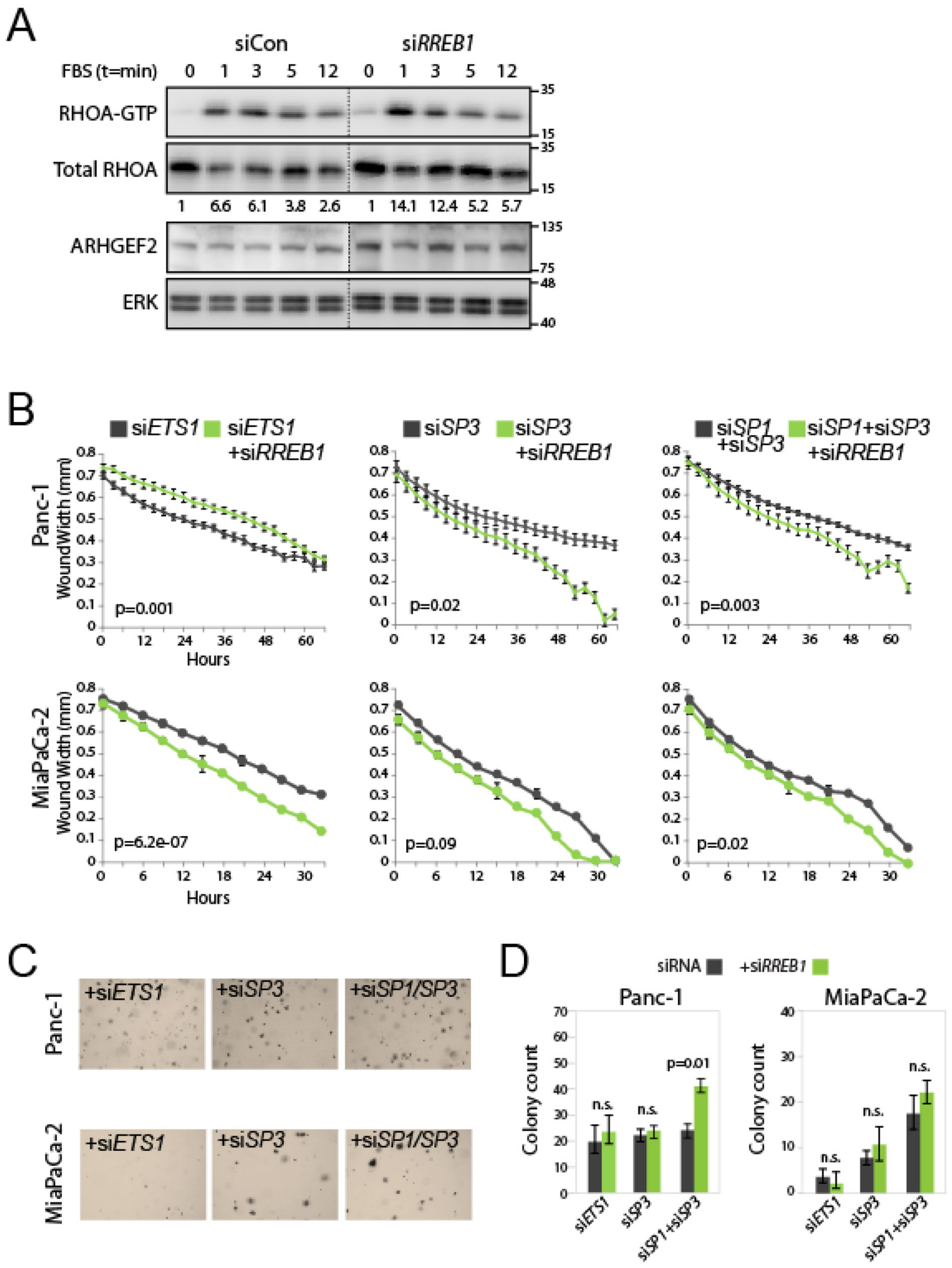

Supplementary Figure S6: A. Western blot analysis of RHOA-GTP expression in Panc-1 cells transfected with siRNA control (siCon) or siRNA targeting RREB1 (siRREB1). 48 hours post siRNA transfection, serum starved cells were stimulated with FBS for the indicated amount of time. Lysates were incubated with Rhotekin-RHO binding domain protein beads, purified and subjected to western blot analysis. The average ratio of RHOA-GTP/total RHOA from three independent experiments is indicated. ERK served as a loading control. **B.** Graphs of wound width (mm) of Panc-1 and MiaPaCa-2 cells transfected with the indicated siRNA (black line) or siRNA plus siRNA targeting *RREB1* (green line) monitored over the indicated time course. P-values calculated from wound width analysis at 40 hour time point (Panc-1) and 18 hour time point (MiaPaCa-2). **C, D.** Representative images (C) and quantification (D) of Panc-1 and MiaPaCa-2 cells transfected with siRNA targeting *RREB1* plus the indicated siRNA and grown for 7 days in 0.3% agar to form colonies. Bar graphs average colony counts from 3 representative images. P-values are indicated.

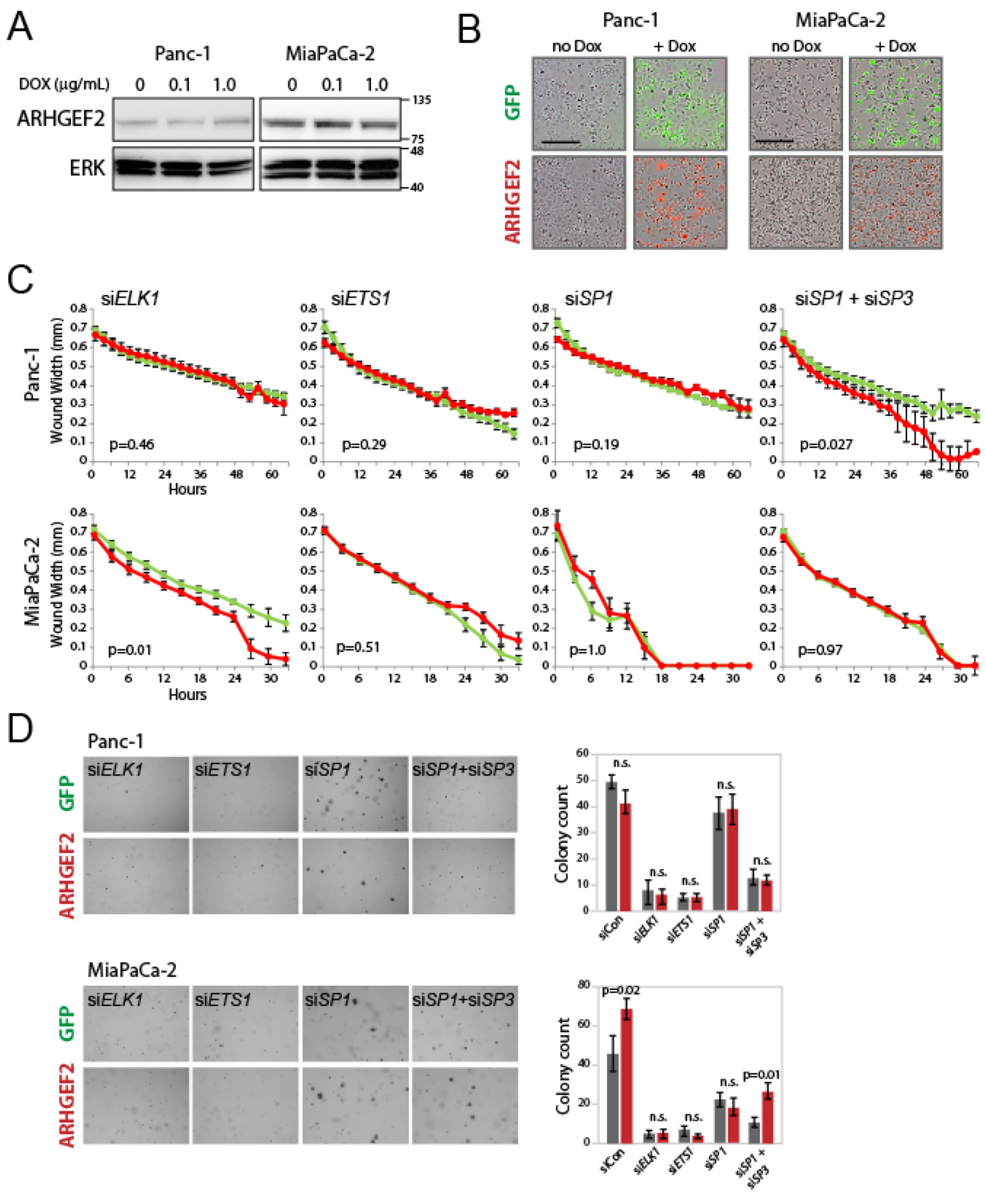

Supplementary Figure S7: A. Western blot analysis of ARHGEF2 protein levels in Panc-1 and MiaPaCa-2 cells containing a doxycycline inducible GFP. Cells were treated with doxycycline and lysates were probed 24 hours post treatment with the indicated antibodies. **B.** Phase contrast images of Panc-1 and MiaPaCa-2 cells containing a doxycycline inducible GFP or cherry-ARHGEF2 in the absence of doxycycline (no Dox) or the presence of doxycycline (+Dox). Bar equals 300 nm. **C.** Graphs of wound width (mm) of GFP expressing (green lines) and cherry-ARHGEF2 expressing (red lines) Panc-1 and MiaPaCa-2 cells transfected with control siRNA (siCon) or the indicated siRNAs monitored over the indicated time course. P-values calculated from wound width analysis at 40 hour time point (Panc-1) and 18 hour time point (MiaPaCa-2). **D.** Representative images and quantification of GFP and cherry-ARHGEF2 expressing Panc-1 and MiaPaCa-2 cells transfected with the indicated siRNA and grown for 7-9 days in 0.3% agar to form colonies. Bar graphs average colony counts from 3 representative images of GFP expressing cells (grey bars) and cherry-ARHGEF2 expressing cells (red bars). P-values are indicated.

Supplementary Table S1: Small molecule compounds used in the study

Compound:	Target(s):	Working Range:	Source:
5Z-7-oxozeaenol	ERK2/TAK1, MEK1	4uM	Sigma
AR-A014414	GSK3a/b	10uM	Sigma
Fasudil	ROCK, TNF-a	20uM	*
IWR-1	Axin	10uM	*
LY2157299	SMAD2	20uM	Selleckchem
LY294002	PI3K	1-5uM	Sigma
RAC1-inhibitor II	RAC1, TIAM1	50uM	Cytoskeleton
SB203580	P38, PKB	40uM	Sigma
SP600125	JNK1/2/3	20uM	Sigma
U0126	MEK1/2	10uM	Sigma
XAV-939	TNKS1/2	10uM	*
Y27632	p160ROCK/ROCK-II	20uM	Sigma
RhoA-Asn-41	RhoA	0.02ug/mL	Cytoskeleton

* Synthesized at OICR

Supplementary Table S2: RHOGEFs and RHOGAPs in the human genome

See Supplementary File 1

Supplementary Table S3: List of primers used in the study

See Supplementary File 1

Supplementary Table S4: Antibodies used in the study

Antibody:	Use:	Source:	Product #:
AKT	Western	Cell Signaling Technologies	9272
Caspase-3	Western	Cell Signaling Technologies	9665
Cleaved caspase-3	Western	Cell Signaling Technologies	9661
ELK1	Western	Cell Signaling Technologies	9182
ELK1	ChIP	Santa Cruz	sc-22804X
ERK	Western	Cell Signaling Technologies	9102
ETS1	Western/ChIP	Santa Cruz	sc-350X
Gef-H1	Western	Ab facility Sunnybrook Hospital Toronto	n/a
KRAS	Western	Santa Cruz	sc-30
pAKT	Western	Cell Signaling Technologies	4060
pERK	Western	Cell Signaling Technologies	9101
RAS	Western	Cell Signaling Technologies	3965
RFP	Western	AbCam	ab-62431
RhoA	Western	Cell Signaling Technologies	2117
RREB1	Western	AbCam	ab-64168
RREB1	ChIP	Sigma Aldrich	HPA001756
SP1	Western/ChIP	Cell Signaling Technologies	9389
SP3	Western/ChIP	Santa Cruz	sc-644
Tubulin	Western	Santa Cruz	sc-69969
