# Supplementary material for: An oncogenic KRAS transcription program activates the RHOGEF ARHGEF2 to mediate transformed phenotypes in pancreatic cancer
Source: Oncotarget. 2016 Nov 7;8(3):4484–500. doi: 10.18632/oncotarget.13152 (PMC5354848; doi:10.18632/oncotarget.13152)
Supplement: Supplementary file 2 [file oncotarget-08-4484-s002.docx]

**Supplemental Table 2: RHOGEFs and RHOGAPs in the human genome.**

| **Ensembl Gene ID** | **Gene Name** | **Group** | **GEF or GAP** |
| --- | --- | --- | --- |
| ENSG00000100852 | ARHGAP5 | 1 | GAP |
| ENSG00000104880 | ARHGEF18 | 1 | GEF |
| ENSG00000105137 | SYDE1 | 1 | GAP |
| ENSG00000106069 | ARHGAP3 (CHN2) | 1 | GAP |
| ENSG00000116584 | ARHGEF2 | 1 | GEF |
| ENSG00000135905 | DOCK10 | 1 | GEF |
| ENSG00000137727 | ARHGAP20 | 1 | GAP |
| ENSG00000163219 | ARHGAP25 | 1 | GAP |
| ENSG00000186716 | BCR | 1 | GEF/GAP |
| ENSG00000198399 | ITSN2 | 1 | GEF |
| ENSG00000058335 | RASGRF1 | 2 | GEF |
| ENSG00000066248 | NGEF | 2 | GEF |
| ENSG00000099331 | MYO9B | 2 | GAP |
| ENSG00000110237 | ARHGEF17 | 2 | GEF |
| ENSG00000122126 | OCRL | 2 | GAP |
| ENSG00000130762 | ARHGEF16 | 2 | GEF |
| ENSG00000132694 | ARHGEF11 | 2 | GEF |
| ENSG00000146426 | TIAM2 | 2 | GEF |
| ENSG00000147251 | DOCK11 | 2 | GEF |
| ENSG00000147799 | ARHGAP39 | 2 | GAP |
| ENSG00000153404 | PLEKHG4B | 2 | GEF |
| ENSG00000159842 | ABR | 2 | GEF/GAP |
| ENSG00000171680 | PLEKHG5 | 2 | GEF |
| ENSG00000205726 | ITSN1 | 2 | GEF |
| ENSG00000240771 | ARHGEF25 (GEFT) | 2 | GEF |
| ENSG00000003393 | ALS2 | 3 | GEF |
| ENSG00000031003 | FAM13B | 3 | GAP |
| ENSG00000035499 | DEPDC1B | 3 | GAP |
| ENSG00000038382 | TRIO | 3 | GEF |
| ENSG00000050327 | ARHGEF5 | 3 | GEF |
| ENSG00000053524 | ARHGEF22 (MCF2L2) | 3 | GEF |
| ENSG00000066933 | MYO9A | 3 | GAP |
| ENSG00000075884 | ARHGAP15 | 3 | GAP |
| ENSG00000076928 | ARHGEF1 | 3 | GEF |
| ENSG00000079482 | OPHN1 | 3 | GAP |
| ENSG00000088538 | DOCK3 | 3 | GEF |
| ENSG00000088756 | ARHGAP28 | 3 | GAP |
| ENSG00000089639 | GMIP | 3 | GAP |
| ENSG00000089820 | ARHGAP4 | 3 | GAP |
| ENSG00000090924 | PLEKHG2 | 3 | GEF |
| ENSG00000100092 | SH3BP1 | 3 | GAP |
| ENSG00000102606 | ARHGEF7 | 3 | GEF |
| ENSG00000105647 | PIK3R2 | 3 | GAP |
| ENSG00000107099 | DOCK8 | 3 | GEF |
| ENSG00000114790 | ARHGEF26 (SGEF) | 3 | GEF |
| ENSG00000120278 | PLEKHG1 | 3 | GEF |
| ENSG00000120318 | ARAP3 | 3 | GAP |
| ENSG00000123329 | ARHGAP9 | 3 | GAP |
| ENSG00000124126 | PREX1 | 3 | GEF |
| ENSG00000124143 | ARHGAP40 | 3 | GAP |
| ENSG00000126217 | MCF2L | 3 | GEF |
| ENSG00000126822 | PLEKHG3 | 3 | GEF |
| ENSG00000128512 | DOCK4 | 3 | GEF |
| ENSG00000129675 | ARHGEF6 | 3 | GEF |
| ENSG00000130158 | DOCK6 | 3 | GEF |
| ENSG00000136002 | ARHGEF4 | 3 | GEF |
| ENSG00000137135 | ARHGEF39 | 3 | GEF |
| ENSG00000138640 | FAM13A | 3 | GAP |
| ENSG00000145675 | PIK3R1 | 3 | GAP |
| ENSG00000152767 | FARP1 | 3 | GEF |
| ENSG00000160007 | ARHGAP35 | 3 | GAP |
| ENSG00000161800 | RACGAP1 | 3 | GAP |
| ENSG00000163486 | SRGAP2B | 3 | GAP |
| ENSG00000163947 | ARHGEF3 | 3 | GEF |
| ENSG00000164741 | ARHGAP7 (DLC1) | 3 | GAP |
| ENSG00000165322 | ARHGAP12 | 3 | GAP |
| ENSG00000165895 | ARHGAP42 | 3 | GAP |
| ENSG00000175220 | ARHGAP1 | 3 | GAP |
| ENSG00000180263 | FGD6 | 3 | GEF |
| ENSG00000180448 | HMHA1 | 3 | GAP |
| ENSG00000182957 | SPATA13 | 3 | GEF |
| ENSG00000186517 | ARHGAP30 | 3 | GAP |
| ENSG00000186635 | ARAP1 | 3 | GAP |
| ENSG00000225485 | ARHGAP23 | 3 | GAP |
| ENSG00000004777 | ARHGAP33 | 4 | GAP |
| ENSG00000006607 | FARP2 | 4 | GEF |
| ENSG00000006740 | ARHGAP44 | 4 | GAP |
| ENSG00000008323 | PLEKHG6 | 4 | GEF |
| ENSG00000017797 | RALBP1 | 4 | GAP |
| ENSG00000024526 | DEPDC1 | 4 | GAP |
| ENSG00000031081 | ARHGAP31 | 4 | GAP |
| ENSG00000046889 | PREX2 | 4 | GEF |
| ENSG00000047365 | ARAP2 | 4 | GAP |
| ENSG00000047648 | ARHGAP6 | 4 | GAP |
| ENSG00000071205 | ARHGAP10 | 4 | GAP |
| ENSG00000074964 | ARHGEF10L | 4 | GEF |
| ENSG00000097096 | SYDE2 | 4 | GAP |
| ENSG00000100485 | SOS2 | 4 | GEF |
| ENSG00000102302 | FGD1 | 4 | GEF |
| ENSG00000104728 | ARHGEF10 | 4 | GEF |
| ENSG00000107554 | DNMBP | 4 | GEF |
| ENSG00000107863 | ARHGAP21 | 4 | GAP |
| ENSG00000113319 | RASGRF2 | 4 | GEF |
| ENSG00000114346 | ECT2 | 4 | GEF |
| ENSG00000115904 | SOS1 | 4 | GEF |
| ENSG00000128805 | ARHGAP22 | 4 | GAP |
| ENSG00000130052 | STARD8 | 4 | GAP |
| ENSG00000131089 | ARHGEF9 | 4 | GEF |
| ENSG00000133121 | STARD13 | 4 | GAP |
| ENSG00000134215 | VAV3 | 4 | GEF |
| ENSG00000134516 | DOCK2 | 4 | GEF |
| ENSG00000137962 | ARHGAP29 | 4 | GAP |
| ENSG00000140750 | ARHGAP17 (RICH1) | 4 | GAP |
| ENSG00000141968 | VAV1 | 4 | GEF |
| ENSG00000142632 | ARHGEF19 | 4 | GEF |
| ENSG00000145819 | ARHGAP26 | 4 | GAP |
| ENSG00000146192 | FGD2 | 4 | GEF |
| ENSG00000146376 | ARHGAP18 | 4 | GAP |
| ENSG00000147256 | ARHGAP36 | 4 | GAP |
| ENSG00000147459 | DOCK5 | 4 | GEF |
| ENSG00000150760 | DOCK1 | 4 | GEF |
| ENSG00000154783 | FGD5 | 4 | GEF |
| ENSG00000156299 | TIAM1 | 4 | GEF |
| ENSG00000159314 | ARHGAP27 | 4 | GAP |
| ENSG00000160145 | KALRN | 4 | GEF |
| ENSG00000160293 | VAV2 | 4 | GEF |
| ENSG00000163486 | SRGAP2 | 4 | GAP |
| ENSG00000164691 | TAGAP | 4 | GAP |
| ENSG00000165801 | ARHGEF40 | 4 | GEF |
| ENSG00000170776 | AKAP13 (ARHGEF13, LBC) | 4 | GEF |
| ENSG00000173848 | ARHGEF8 (NET1) | 4 | GEF |
| ENSG00000183111 | ARHGEF37 | 4 | GEF |
| ENSG00000187951 | ARHGAP11B | 4 | GAP |
| ENSG00000196155 | PLEKHG4 | 4 | GEF |
| ENSG00000196220 | SRGAP3 | 4 | GAP |
| ENSG00000196914 | ARHGEF12 | 4 | GEF |
| ENSG00000196935 | SRGAP1 | 4 | GAP |
| ENSG00000198826 | ARHGAP11A | 4 | GAP |
| ENSG00000198844 | ARHGEF15 | 4 | GEF |
| ENSG00000203734 | ECT2L | 4 | GEF |
| ENSG00000204084 | INPP5B | 4 | GAP |
| ENSG00000213390 | ARHGAP19 | 4 | GAP |
| ENSG00000214694 | ARHGEF33 | 4 | GEF |
| ENSG00000214944 | ARHGEF28 (RGNEF) | 4 | GEF |
| ENSG00000236699 | ARHGEF38 | 4 | GEF |
| ENSG00000241484 | ARHGAP8 | 4 | GAP |
| ENSG00000088387 | DOCK9 | 5 | GEF |
| ENSG00000101977 | MCF2 | 5 | GEF |
| ENSG00000116641 | DOCK7 | 5 | GEF |
| ENSG00000127084 | FGD3 | 5 | GEF |
| ENSG00000128656 | ARHGAP2 (CHN1) | 5 | GAP |
| ENSG00000134909 | ARHGAP32 | 5 | GAP |
| ENSG00000138639 | ARHGAP24 | 5 | GAP |
| ENSG00000139132 | FGD4 | 5 | GEF |
| ENSG00000154358 | OBSCN | 5 | GEF |
| ENSG00000187510 | PLEKHG7 | 5 | GEF |
|  |  |  |  |

**Supplemental Table 3: List of primers used in the study.**

| **1. QPCR primers:** |  |  |
| --- | --- | --- |
| GENE: | F: | R: |
| ARHGEF2 | CAGGCATGACCATGTGCTATG | TTTACAGCGGTTGTGGATAGTC |
| BACT | AGGCACCAGGGCGTGAT | GCCCACATAGGAATCCTTCTGAC |
| ELK1 | TAACAGACACCTCTGGCTG | AATTCAAGCTGGTGGATGC |
| ETS1 | TCATTTCTTTGCTGCTTGGA | CTCACCATCATCAAGACGGA |
| Fos | GTGGGAATGAAGTTGGCACT | ACGGATCTATACCAACGCCA |
| ITSN2 | AGGTGTTGGAAACTGAGCCA | GTGAGCGGCACTGATTTGT |
| Jun | CCCCCAGCGTATCTATATGGAA | GCTGTCCCTCTCCACTGCAA |
| KLF5 | TCCCAGGTACACTTGTATGGC | ACCCTGGTTGCACAAAAGTT |
| MZF1 | GAGGGCTCCATCTTCTCTGA | GAGGCTGCTGCCCTAGTAGA |
| RREB1 | CCTCTGAAACGTAGGCGATTGT | GGCATCGTGACTCAGTTTCCTC |
| SP1 | GGCACAAACGTACACACACA | CTGGGCCTCCCTTCTTATTC |
| SP3 | AGAATCACCACTGAGCTGCC | CATCCAGGAGAGAATGCTGAC |
| Spi1 | GGGGTGGAAGTCCCAGTAAT | ACGGATCTATACCAACGCCA |
| STAT1 | CCATCCTTTGGTACAACATGC | TGCACATGGTGGAGTCAGG |
| STAT3 | GATCCAGTCCGTGGAACCAT | ATAGCCCATGATGATTTCAGCAA |
|  |  |  |
| **2. ChIP primers:** | F: | R: |
| TSS ChIP ARHGEF2 | GTCTCGGGGACAGGAAGTCT | CTACCCCCTTCCTGAGCTTG |
| 500bpDN ARHGEF2 | AGCGATTGGGGAGTGATG | AGAGGGAGGTGTGTGTCTCG |
| 500bpUP ARHGEF2 | ACGGGAAGGGTAGGATTCAC | AGCTAGGAGGAGGGCAGAGT |
|  |  |  |
| **3. Promoter cloning primers:** |  |  |
| Promoter (see Fig2) | F: | R: |
| AP6 | GCGGAGATCTAAAGGAGCCCAGCTACACAA | GCGGCTAGCCTGCTAACCGAAGGCTCTGT |
| AP8 | GCGGAGATCTAAAGGAGCCCAGCTACACAA | GCGGCTAGCctacccccttcctgagcttg |
| AP9 | GCGGAGATCtacccactcgctcgcagtc | GCGGCTAGCCTGCTAACCGAAGGCTCTGT |
| AP10 | GCGGAGATCtacccactcgctcgcagtc | GCGGCTAGCaaaaggggtcagtctgctca |
| AP10.5 | GCGGAGATCTCTCTGCTCCGGTCGAT | GCGGCTAGCGGGTTTTAGGAAAAGAAGAGCTAA |
| AP11 | GCGGAGATCtacccactcgctcgcagtc | GCGGCTAGCctacccccttcctgagcttg |
| AP12 | GCGGAGATCTCCCGCCTCTTAGCTCTTCTT | GCGGCTAGCCTGCTAACCGAAGGCTCTGT |
| AP13 | GCGGAGATCtacccactcgctcgcagtc | GCGGCTAGCGGGTTTTAGGAAAAGAAGAGCTAA |
| AP14 | GCGGAGATCTCTCTGCTCCGGTCGAT | GCGGCTAGCctacccccttcctgagcttg |
| AP15 | GCGGAGATCTGTCTCGGGGACAGGAAGTCT | GCGGCTAGCGGGTTTTAGGAAAAGAAGAGCTAA |
| AP16 | GCGGAGATCTGTCTCGGGGACAGGAAGTCT | GCGGCTAGCCTGCTAACCGAAGGCTCTGT |
| AP17 | GCGGAGATCTGTCTCGGGGACAGGAAGTCT | GCGGCTAGCaaaaggggtcagtctgctca |
|  |  |  |
| **4. Mutagenesis primers:** |  |  |
| TF binding site: | F: | R: |
| AP1 | ccgggcgagggttttcagacttcctgtccccgaga | Tctcggggacaggaagtctgaaaaccctcgcccgg |
| ELK1/ETS1 | Cacgcgttggtctcggtgaaagtacgtccgactcccctcgcccgg | Ccgggcgaggggagtcggacgtactttcaccgagaccaacgcgtg |
| MZF1 (#1) | caggaagtctgactcttcacgcccggcacccagc | gctgggtgccgggcgtgaagagtcagacttcctg |
| MZF1 (#2) | ggacaggtttgagaggcttcactgcctatttcccctcc | ggaggggaaataggcagtgaagcctctcaaacctgtcc |
| RREB1 | acgggacgccaggcttcgtctgcgggcagctcggag | Ctccgagctgcccgcagacgaagcctggcgtcccgt |
| SP1 (#3) | Tgcgcgcgggcaccttgttctccctccgctccg | cggagcggagggagaacaaggtgcccgcgcgca |
| Spi1 (#1) | ggcacccagcaccaggacgtcaaacccgagtctc | gagactcgggtttgacgtcctggtgctgggtgcc |
| Spi1 (#2) | gcctacccccagcatgagcttgggcgggggagg | cctcccccgcccaagctcatgctgggggtaggc |
| STAT (#1) | Ggcacccagcaccagttactcgagcacgagtctcctccccc | Gggggaggagactcgtgctcgagtaactggtgctgggtgcc |
| STAT (#2) | gagctcttacgcgtgctagcgggcctcaagccaagaagagctaagaggcgggtct | agacccgcctcttagctcttcttggcttgaggcccgctagcacgcgtaagagctc |
|  |  |  |
